# Atomic-level characterization of transport cycle thermodynamics in the glycerol-3-phosphate:phosphate antiporter

**DOI:** 10.1038/ncomms9393

**Published:** 2015-09-29

**Authors:** Mahmoud Moradi, Giray Enkavi, Emad Tajkhorshid

**Affiliations:** 1 Department of Biochemistry, Beckman Institute for Advanced Science and Technology, and Center for Biophysics and Computational Biology, University of Illinois at Urbana-Champaign, Urbana, Illinois 61801, USA

## Abstract

Membrane transporters actively translocate their substrate by undergoing large-scale structural transitions between inward- (IF) and outward-facing (OF) states (‘alternating-access' mechanism). Despite extensive structural studies, atomic-level mechanistic details of such structural transitions, and as importantly, their coupling to chemical events supplying the energy, remain amongst the most elusive aspects of the function of these proteins. Here we present a quantitative, atomic-level description of the functional thermodynamic cycle for the glycerol-3-phosphate:phosphate antiporter GlpT by using a novel approach in reconstructing the free energy landscape governing the IF↔OF transition along a cyclic transition pathway involving both *apo* and substrate-bound states. Our results provide a fully atomic description of the complete transport process, offering a structural model for the alternating-access mechanism and substantiating the close coupling between global structural transitions and local chemical events.

## Abstract (web summary)

Membrane transporters transition between inward- and outward-facing conformations to translocate substrates across membranes but mechanistic details of this process remain scarce. Here Moradi *et al*. use an advanced molecular simulation approach to describe the complete thermodynamic cycle of the GlpT transporter at atomic-level detail.

## Graphical abstract

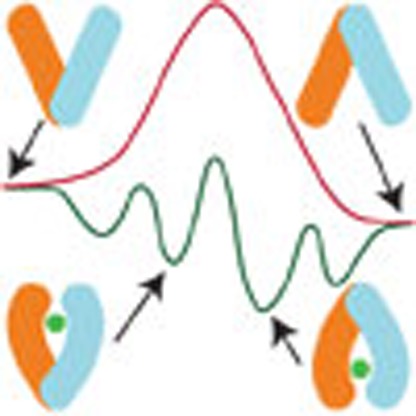

Membrane transporters are primary cellular gatekeepers that carry out active exchange of materials in and out of the cell using diverse sources of biochemical energy. Their central role in such essential physiological processes as neurotransmission and metabolism, and direct implication in a wide array of pathophysiological conditions have rendered them as a major class of drug targets^1^.

Major facilitator superfamily (MFS) transporters^2^ constitute the largest superfamily of secondary transporters including several biomedically important proteins involved in pathophysiology of various diseases such as diabetes mellitus^3^ and antibiotic resistance^4^. Highlighted as a structural and functional model for MFS transporters^5^,^6^,^7^, glycerol-3-phosphate (G3P) transporter (GlpT)^8^ manifests the common MFS topology and consists of two bundles of transmembrane (TM) helices with pseudo-twofold symmetry (that is, the N- (NTD) and C-terminal (CTD) halves including TM helices H1–H6 and H7–H12, respectively)^5^,^8^,^9^. Inferred from the only available crystal structure of GlpT in the IF state^8^, a ∼10° rigid-body rotation of the two halves has been proposed to open the periplasmic side and close the cytoplasmic side, thereby generating the OF state^5^,^8^,^10^,^11^.

Under physiological conditions, GlpT facilitates uphill import of G3P using downhill export of inorganic phosphate (P_i_)^12^; Cytoplasmic binding of P_i_ to the IF state presumably facilitates the IF→OF transition^5^,^8^,^10^, while replacement of P_i_ by G3P on the periplasmic side induces the back transition of GlpT to the IF state through a reverse set of protein conformational changes^8^,^5^,^10^. In the absence of organic phosphates, GlpT mediates P_i_:P_i_ exchange^13^, an observation that is exploited here to simplify the study of the transport mechanism. Kinetic studies have shown that the rate-limiting step in the transport cycle is the IF↔OF conformational change, which is accelerated by substrate binding^14^. Based on the crystal structure^8^ and previous biochemical and computational studies^14^,^15^,^16^,^17^, a putative binding site for the IF state has been established; however, the OF state and, more importantly, conformational changes of the protein and the binding site during the IF↔OF transition remain elusive.

The scope of atomistic simulations, which are required for proper description of transport mechanism, is often limited to local conformational dynamics of individual states with known structures, unless enhanced sampling techniques are employed^18^. We have recently introduced an efficient computational framework for the study of large-scale conformational transitions, without compromising important atomic details, using a combination of several distinct enhanced sampling techniques^19^,^20^, further developed here into a practical approach for the study of membrane transporters.

A conformational transition pathway is reconstructed via iterative molecular dynamics (MD) simulations aimed at improving the sampling by using progressively more optimal reaction coordinates and more relaxed conformations. The main sampling tool used here is the bias-exchange umbrella sampling (BEUS)^19^,^20^,^21^. The success of the method when applied to complex structural transitions, however, relies on an extensive empirical search for mechanistically relevant collective variables using a large number of exploratory, short nonequilibrium simulations during the initial phase^19^,^20^. For relaxation of the pathways in high-dimensional collective variable spaces, we use a parallel implementation of string method with swarms of trajectories (SMwST)^22^. We use this method to further refine putative transition pathways that are generated by employing yet another path-finding algorithm, developed in this study, that is, *post-hoc* string method (PHSM), an analysis technique to extract an approximate minimum free energy path (or principal curve^23^) from prior simulations. The PHSM algorithm plays a pivotal role in our iterative approach by allowing to extract, in an efficient manner, the most relevant set of conformations from prior BEUS simulation(s) to initiate a SMwST or another BEUS simulation.

Employing the novel approach discussed above, we have conducted an extensive computational study of the entire thermodynamic cycle of GlpT, which is aimed at describing not only the unknown OF state but also the entire conformational transition pathway between the IF and OF states both in the absence (*apo*) and in the presence (bound) of the transport substrate. Our results provide a detailed view of the large-scale conformational changes of GlpT that are closely coupled to chemical events within the lumen, specifically binding, translocation and unbinding of the substrate, suggesting that a complex, dynamic sequence of substrate-protein interactions is involved in the transport process. In particular, our results indicate state-specific substrate-binding modes for GlpT involving distinct binding residues in the IF and OF conformations. The binding site configuration thus undergoes a significant change during the IF↔OF transition. We observe distinct structural transition pathways for substrate-bound and *apo* transporters indicating a central role for the substrate in the conformational transition mechanism of GlpT. These findings are in line with both alternating-access and rocker-switch mechanisms, although they suggest that the rocker-switch motion of the NTD and CTD bundles is accompanied by a relative twist of the two domains which represents a major mechanistic difference between the substrate-bound and *apo* IF↔OF transitions.

## Results

### Reconstructing the transport cycle

**Figure 1:**
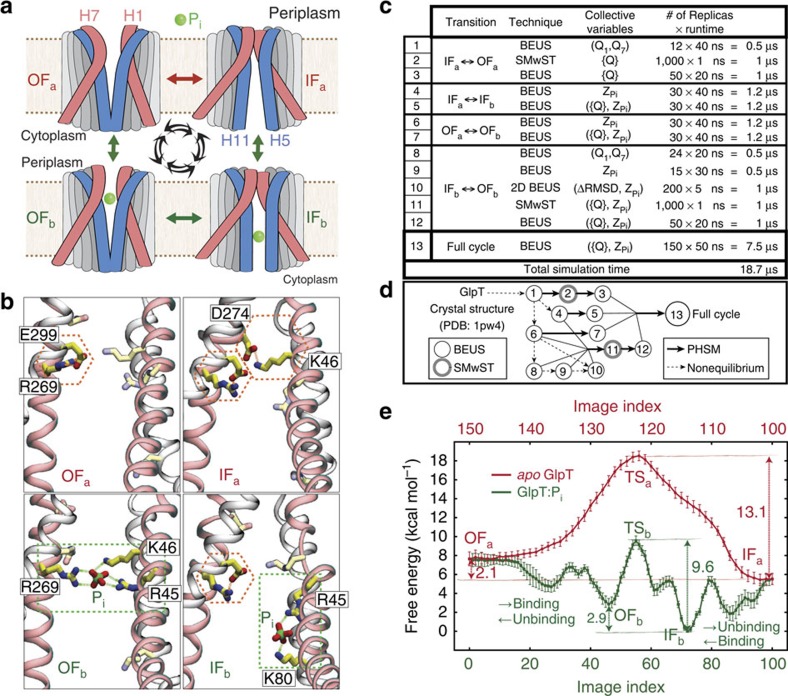
Thermodynamic cycle of GlpT:P_i_ complex. (**a**) Schematic representation of the states involved in the thermodynamic cycle under study. TM helices H1/H7 (red) and H5/H11 (blue) are highlighted as representative helices involved in peri- and cytoplasmic gating, respectively. (**b**) Luminal charged residues involved in substrate binding (green boxes) and/or salt bridge formation (red hexagons). TM helices H1/H7 (red) and H2/H8 (grey) are shown. (**c**) List of the BEUS and SMwST simulations conducted to reconstruct the full thermodynamic cycle shown in **a**. Each replica consists of a fully atomic GlpT protein in an explicit membrane/water environment including ∼125,000 atoms. (**d**) Graph showing the iterative scheme used for designing the simulations shown in **c**. (**e**) Free energy profile along the simulated thermodynamic cycle shown in **a** based on the final simulation (set 13), involving both *apo* and bound GlpT. Each image (or window) represents a particular conformation of GlpT:P_i_ complex on a discretized cyclic transition pathway composed of 150 images defined in the ({*Q*},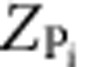) space. The error bars represent the s.d. based on Bayesian block bootstrapping (see Analysis techniques in Methods section).

The simulated thermodynamic cycle of GlpT involves four distinct functional states: OF-*apo*, OF-bound, IF-bound and IF-*apo*, denoted as OF_*a*_, OF_*b*_, IF_*b*_ and IF_*a*_, respectively (Fig. 1a,b; also see Supplementary Movie 1). Starting from the IF_*a*_ state, modelled based on the only available crystal structure of GlpT^8^, we set out to describe the transitions involved in the thermodynamic cycle shown in Fig. 1a. To design a sampling protocol that simultaneously takes into account the concerted motion of the protein and substrate binding/translocation/unbinding (which involves multiple binding residues as well as the substrate), we conducted an extensive set of simulations illustrated in Fig. 1c,d (and Supplementary Table 1) and discussed in detail in Methods section and Supplementary Note 1.

The task of finding the OF state was first done using *apo* GlpT simulations employing an empirical search for efficient biasing protocols^20^. The optimum protocol to generate the OF_*a*_ state (Supplementary Fig. 1a) involved imposing rotational changes on TM helices H1 and H7, followed by free energy calculations along the optimized IF_*a*_↔OF_*a*_ transition pathway (Fig. 1c: simulation sets 1–3). In the second stage, two independent sets of free energy calculations were performed in the presence of P_i_, starting from the equilibrated IF_*a*_ and OF_*a*_ states to characterize the substrate binding/unbinding process, and to identify the IF_*b*_ and OF_*b*_ states (simulation sets 4/5 and 6/7, respectively). These two bound states were found to be distinct, not only in terms of the protein global conformation but, crucially, also in their local substrate-binding site configuration. The IF_*b*_↔OF_*b*_ transition involves a ∼8-Å translocation of the substrate within the lumen along the *z* axis that requires significant local conformational changes within the biding site. Owing to its complexity, IF_*b*_↔OF_*b*_ transition was thus studied using multiple sets of free energy calculations and path-finding algorithms in an iterative manner (simulation sets 8, 9, 10, 11 and 12).

**Figure 2:**
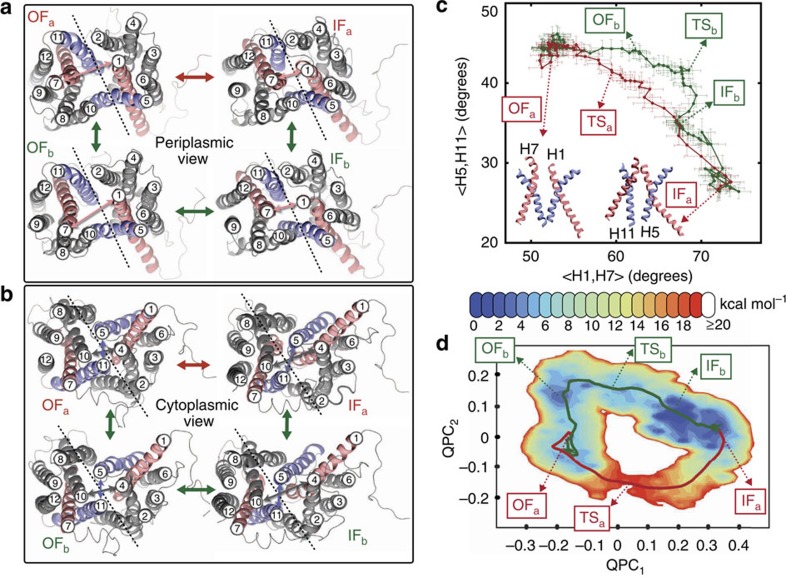
Global conformational changes of GlpT. (**a**) Peri- and (**b**) cytoplasmic views of GlpT in OF_*a*_, OF_*b*_, IF_*b*_ and IF_*a*_ states. The dashed lines approximately represent the axis/plane of twofold pseudosymmetry. TM helices H1/H7 and H5/H11 are coloured red and blue, respectively. (**c**) Interhelical angle of H5/H11 helices plotted against that of H1/H7 helices. Here <A,B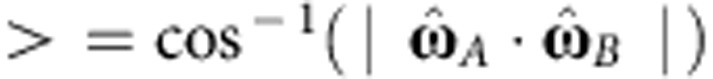, in which **ω** is the roll axis of the helix (obtained from principal axis component analysis based on C^*α*^ atoms). <H1,H7> and <H5,H11> approximately represent the global conformational changes leading to the opening/closing of the peri- and cytoplasmic sides, respectively. The error bars represent the s.d. (see Analysis techniques in Methods section). (**d**) PMF in terms of (*QPC*_1_,*QPC*_2_), that is, the first two principal components of the vector parts of the {*Q*} quaternions. The pathways shown in **c** and **d** are based on the (unweighted) averaged centres of the 150 images (also see Fig. 1e).

**Figure 3:**
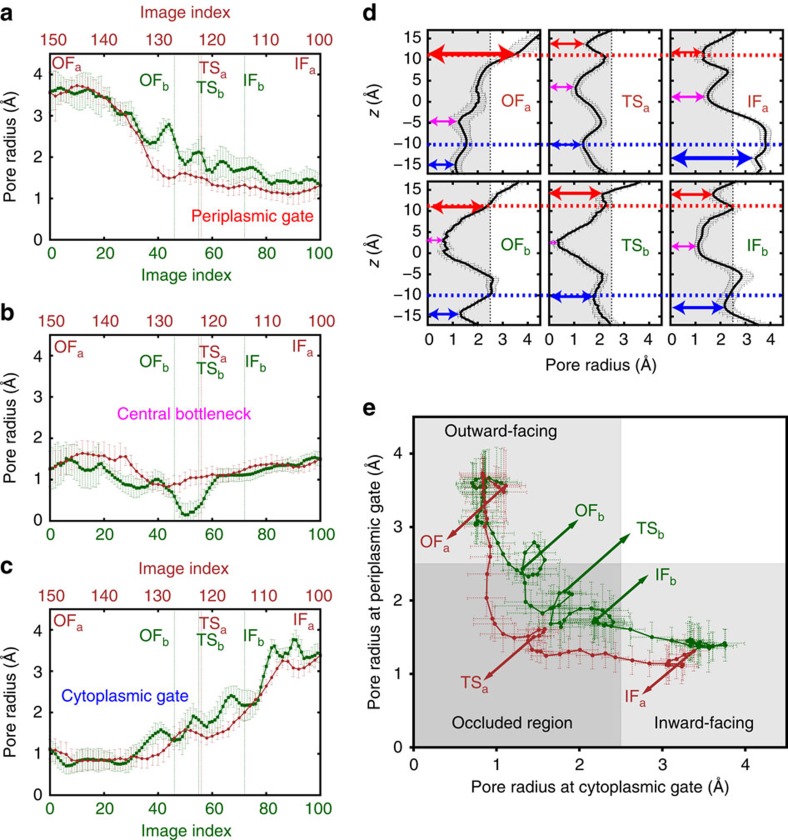
Alternating-access mechanism. Pore radius at (**a**) periplasmic gate, (**b**) central bottleneck and (**c**) cytoplasmic gate along 150 simulated images of GlpT, averaged over sampled conformations of each image (see Supplementary Fig. 7 for the definitions and the lists of most frequently involved residues). The pore radius along the *z* axis (membrane normal) of each sampled conformation was measured with HOLE^59^ using protein and P_i_ atoms. (**d**) Pore radius along *z* axis shown for a select number of images. The arrows indicate the peri- (red) and cytoplasmic (blue) gates as well as the central bottleneck (magenta) used in panels **a**–**c**. (**e**) Pore radius at peri- and cytoplasmic gates. In both *apo* and bound GlpT, the IF↔OF transition occurs by visiting an occluded region in which both gates are closed to the substrate. The error bars represent the s.d. (see Analysis techniques in Methods section).

**Figure 4:**
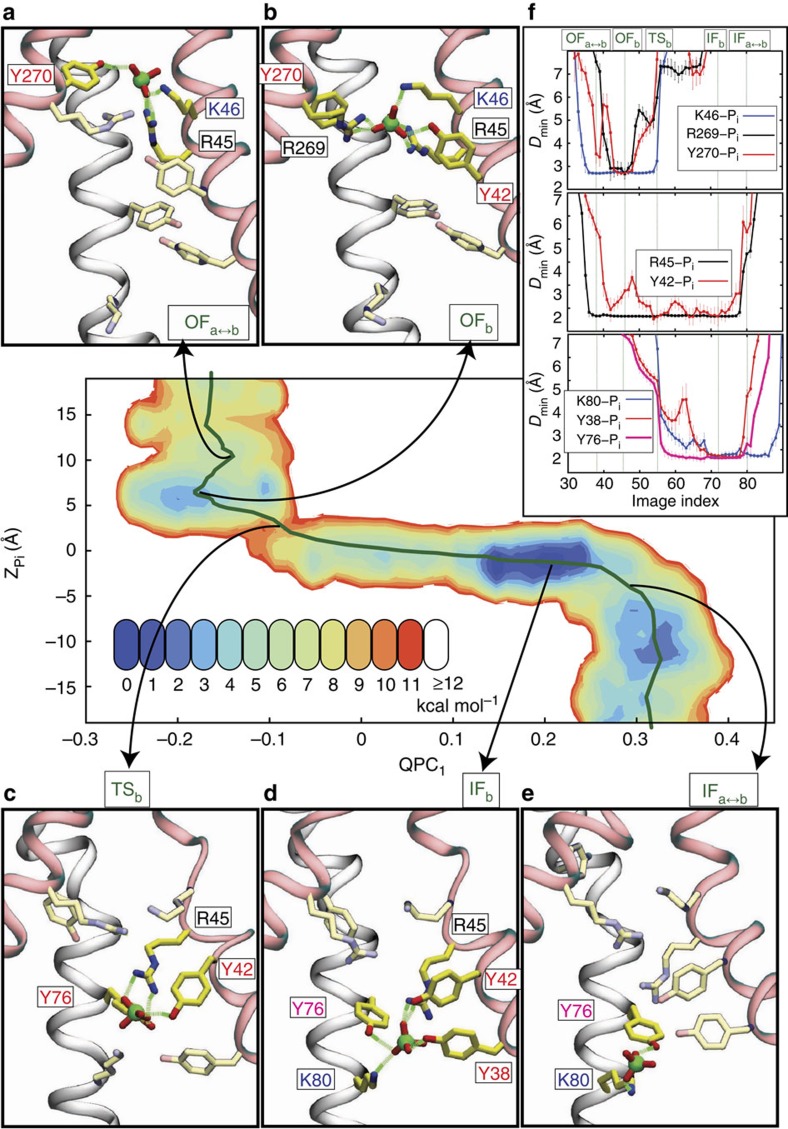
Substrate binding, translocation and unbinding coupled to protein conformational changes. PMF in the (*QPC*_1_,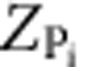) space is shown for the regions in which P_i_ is present in the lumen. (**a**–**e**) Snapshots of the binding site conformation at a select number of images representing the early stages of binding/unbinding in the peri- (**a**) and cytoplasmic (**e**) sides, the fully-bound OF_*b*_ (**b**) and IF_*b*_ (**d**), and the TS_*b*_ transition state **c**. (**f**) *D*_min_ (minimum hydrogen donor-acceptor distance) between the P_i_ and side chains shown in **a**–**e**, grouped into three classes based on the functional state in which they interact with the substrate: OF state (top panel), IF state (bottom panel) and both states (middle panel). The residues shown in **a**–**e** are those strongly interacting with P_i_ (defined by *D*_min_<3 Å) at least at one image. *D*_min_ of each image is an unweighted average over all sampled conformations of the image. The error bars represent the s.d. (see Analysis techniques in Methods section).

Combining the optimum OF_*a*_↔OF_*b*_, OF_*b*_↔IF_*b*_, IF_*b*_↔IF_*a*_ and IF_*a*_↔OF_*a*_ transition pathways obtained from simulation sets 1–12, a closed curve in the ({*Q*}, 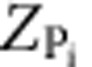) space was built, representing a ‘cyclic' pathway connecting OF_*a*_, OF_*b*_, IF_*b*_ and IF_*a*_ states of the GlpT:P_i_ complex. Here 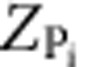 represents the P_i_ position along the membrane normal tracking the substrate binding, translocation and unbinding events, and {*Q*} is a multidimensional collective variable that describes the global conformation of the TM helices based on their orientation quaternions^19^,^20^. The cyclic transition pathway was discretized into 150 images (or windows) and sampled together in a single massive run (simulation set 13), to generate the results presented in Figs 1, 2, 3, 4, Supplementary Figs 2–8 and 10 and Supplementary Tables 2 and 3. In addition, Supplementary Figs 1 and 9 show some of the results obtained from unbiased or nonequilibrium simulations not listed in Fig. 1c, while Supplementary Figs 11–13 illustrate some of the results from simulations sets 1–12.

### Transport cycle thermodynamics.

The close substrate-protein cooperation towards facilitating the transport process is noticeable. While the transporter is a necessary element for catalysing the transport reaction, the substrate itself is needed to facilitate the conformational changes required for the process. The full-cycle free energy profile (Fig. 1d; also see Supplementary Movie 1) is reminiscent of a catalytic reaction, in which the substrate facilitates the IF–OF interconversion of the transporter. This picture suggests an obligatory exchange mechanism in which the rates for *apo* transitions are considerably lower than the corresponding rates in the substrate-bound form (as qualitatively assessed from the free energy barriers). For instance, during the IF_*a*_→OF_*a*_ transition, substrate binding lowers the barrier by 3.52±0.92 kcal mol^−1^ (mean±s.d.; see Analysis techniques in Methods section for details), thereby facilitating the interconversion of the two states. The free energy of the OF state is 2.05±0.88 (2.88±0.59) kcal mol^−1^ higher than that of the IF state in the *apo* (P_i_-bound) GlpT—which is consistent with the fact that GlpT crystallizes favourably in the IF state^8^ (assuming crystal contacts have not altered the resting state conformation). The 0.82±1.06 kcal mol^−1^ shift in the IF–OF free energy difference between the *apo*- and P_i_-bound GlpT (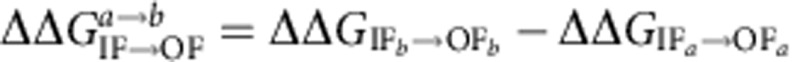) is not as significant as the shift in the transition barrier. Similarly, substrate-binding free energies of the IF and OF states (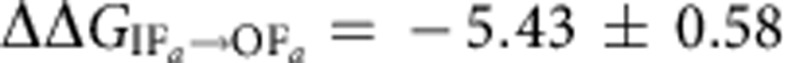 and 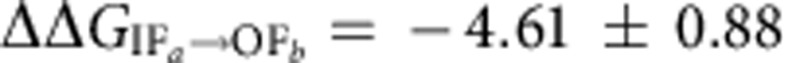 kcal mol^−1^, respectively) are not significantly different. We note that the free energy differences are reported based on the states determined at the corresponding free energy extrema (that is, images 5, 46, 55, 72, 102 and 122 for OF_*a*_, OF_*b*_, TS_*b*_, IF_*b*_, IF_*a*_ and TS_*a*_, respectively), which do not take into account the shape of the free energy landscape. In addition, certain terms such as the entropic gain of ‘free' substrate in the bulk are ignored in our binding free energy calculations. The phosphate is constricted in all simulations to stay within a cylinder centred along the pore. All *apo* free energies thus must be shifted down by ∼0.1 kcal mol^−1^ (as estimated based on the protocol discussed in ref. 24). A more rigorous treatment is however necessary to calculate the standard free energy of binding^25^.

### Global protein conformational changes

With a putative transition pathway which, due to extensive sampling, is relaxed and reversible, we can characterize both global and local protein conformational changes along the pathway and substantiate the mechanism of their coupling. Figure 2, for instance, shows how the peri- and cytoplasmic conformational changes are coupled (also see Supplementary Fig. 2). TM helices H1 and H7 are directly involved in the opening/closing (gating) of the periplasmic side while TM helices H4, H5, H10 and H11 are more directly involved in gating of the cytoplasmic side. Relative rotational change of H1 and H7 helices along the cycle is plotted against that of H5 and H11 helices in Fig. 2c (also see Supplementary Fig. 3). In the *apo* simulations, these angles are correlated almost linearly indicating a coupling between the opening/closing of the peri- and cytoplasmic sides, a hallmark of the proposed rocker-switch mechanism in GlpT^8^. In the P_i_-bound simulations, the behaviour deviates from an ideal rocker-switch model. Note that the interhelical angles are defined based on the roll axes of the helices and may not fully capture their complex nonlinear behaviour (for example, H7 kinking as illustrated in Supplementary Fig. 3 for example; also see ref. 26). Nonetheless, these interhelical angles provide an effective representation of the substrate-induced global structural changes of GlpT similar to those reported recently for GlpT homologues^27^,^28^. The motion of these helices is also accompanied by local conformational changes within the binding site. For instance, K46 and D274 (on H1 and H7, respectively) form a strong salt bridge in the IF_*a*_ state which is absent in the other states (Fig. 1b and Supplementary Fig. 3d); therefore, the formation/breakage of this salt bridge may contribute to the relatively slow rate of the IF_*a*_↔OF_*a*_ transition as compared with the IF_*b*_↔OF_*b*_ transition. For the behaviour of the other salt bridges within the lumen see Supplementary Fig. 4.

To track the concerted movement of the TM helices, {*Q*} was projected onto its principal components. The first two components, *QPC*_1_ and *QPC*_2_, collectively account for ∼78% of the variance in {*Q*} of all sampled conformations (Supplementary Fig. 5). Having a large ensemble of ‘reweighted' samples, we can reconstruct the potential of mean force (PMF) in a low-dimensional space such as (*QPC*_1_,*QPC*_2_) as shown in Fig. 2d, which clearly illustrates how the *apo* and P_i_-bound GlpT states take different pathways during the IF↔OF transition. *QPC*_1_ and *QPC*_2_ also provide information the common and different aspects of the concerted conformational changes along the two transition pathways, respectively. *QPC*_1_ roughly describes the concerted motion of the NTD and CTD domains in the opposite directions perpendicular to their pseudosymmetry plane, while *QPC*_2_ describes a motion parallel to this plane (Supplementary Fig. 5). Change in *QPC*_1_ results in a rocker-switch type motion of NTD and CTD bundles, which is accompanied by a twisting motion of the two domains if *QPC*_2_ changes as well. While during the *apo* IF↔OF transition, the change in the (*QPC*_1_,*QPC*_2_) space is relatively smooth, resembling a rocker-switch type movement combined with a slight twisting motion, the P_i_-mediated IF↔OF transition is stage-wise involving distinct motions induced by P_i_ binding/unbinding in the cyto- and periplasmic sides. For an RMSD-based analysis of global conformational changes of GlpT see Supplementary Fig. 6 and Supplementary Table 3. Given the involvement of distinct IF↔OF transition pathways for the substrate bound and *apo* (as clearly illustrated in Fig. 2d), one may postulate a substrate-specific mechanism for IF↔OF transition, for example, for different organic and inorganic phosphates—in line with recent observations for another MFS transporter^29^.

### Alternating-access mechanism

The global structural transitions of GlpT are coupled to mechanistically relevant localized events within the lumen, most importantly to gating motions that control binding site accessibility. As a proxy for substrate accessibility, we monitored the pore radius (and hydration) along the lumen to characterize the details of the alternating-access mechanism (Fig. 3; also see Supplementary Figs 7 and 8 and Supplementary Movie 1). In addition to the peri- and cytoplasmic constriction regions where gating takes place, a central narrow region, termed ‘central bottleneck', was also identified within the lumen, which, interestingly, is persistent even in the absence of the substrate. While monitoring the orientation of TM helices during the transition (Fig. 2c) indicates a rocker-switch mode^8^ of conformational change for *apo* GlpT, examining the luminal radius profile (Fig. 3e) suggests an almost ideal sequential gating mode^30^, thereby revealing that both global and side-chain conformational changes are involved in the process, and the rocker-switch and sequential gating models are consistent.

During the P_i_-bound IF↔OF transition, which is of greater functional relevance, the behaviour of the system deviates from that described by either ideal model (as also reported recently for a GlpT homolog^30^). Substrate binding from either side of the membrane results in a conformation occluded from both sides, which remains occluded to substrate during the entire IF_*b*_↔OF_*b*_ transition. The IF_*b*_ and OF_*b*_ states along with all the conformations visited during the IF_*b*_↔OF_*b*_ transition, therefore, form a relatively large ‘occluded region' (in contrast to a narrowly populated single state) in the conformational space, thereby ensuring an effective alternating-access mechanism (Fig. 3e). Note that some of the conformations occluded to substrate could be permeable to water; however, the leakiness of transporters such as GlpT to water does not interfere with the alternating-access mechanism required for their function^31^.

### Coupling between global and local conformational changes

Figure 4 illustrates the coupling between substrate translocation and global conformational changes of the protein, along with the luminal residues that interact most strongly with the substrate. R45 is the main binding residue in both IF- and OF-bound states and retains its contact with the substrate during the IF_*b*_↔OF_*b*_ transition. This observation is consistent with the experimental results that R45K mutation abrogates both substrate binding and transport^15^. Employing a nonequilibrium alchemical free energy calculation scheme^32^,^33^, we quantified the free energy changes of four major simulated states (OF_*a*_, OF_*b*_, IF_*b*_ and IF_*a*_) due to the R45K mutation. The change in the binding free energy of GlpT due to mutation was estimated to be 4.40±0.89 kcal mol^−1^ (mean±s.d.; see Nonequilibrium alchemical free energy calculations in Methods section for details), which suggests a significant decrease in binding affinity for P_i_ (Supplementary Fig. 9). Our alchemical free energy calculations similarly indicate a substantial increase in conformational free energy difference of IF and OF states (5.45±0.62 and 3.25±0.87 kcal mol^−1^ for *apo* and bound states; Supplementary Fig. 9c,f) due to the R45K mutation. Note that the presence of a high-energy OF state in the transport cycle can impair the transport even if the substrate binds to the protein. While R45K is involved in binding in both IF and OF states, residues K46/R269 are involved only in OF binding and K80 only in IF binding. These results substantiate the experimental observations that R45K mutation completely blocks substrate binding while K80A, R269K or K46L mutations only diminish binding^15^. Interestingly, our simulations reveal that the direct involvement of these residues (except for R45) with the substrate occurs at different stages of the transport cycle, that is, at distinct functional states. Therefore, IF_*b*_ and OF_*b*_ states are characterized not only with distinct global structures but also with distinct local binding configurations. Such state-specific substrate-binding modes can be highlighted, for example, by a ∼8-Å displacement of the substrate within the lumen along the *z* axis during the IF_*b*_↔OF_*b*_ transition (Fig. 1b). In addition to the charged residues, several tyrosine side chains make contact with P_i_; Y270 and Y38 strongly interact with the substrate in the OF_*b*_ and IF_*b*_ states, respectively, while binding/unbinding of Y76 appear to coincide with crossing of the IF–OF transition state. H165 is another residue that interacts with the substrate, particularly around the transition state (Supplementary Fig. 3h). H165 has been proposed to play an important functional role since its mutation to a proline severely affects both binding and transport^15^. Our analysis of H165 reveals that this residue undergoes a conformational change around the IF–OF transition state, which is clearly detectable by measuring its side-chain dihedral angle *χ*_1_ revealing that H165 side-chain flips around the IF–OF transition state in both *apo* and bound states (Supplementary Fig. 3f,g). Although previous MD simulations, based on the IF conformation only, have indicated that H165 protonation can tighten substrate binding^15^, our post-simulation analysis based on the entire transport cycle (using both PROPKA 3.1 (refs 34, 35) and MolProbity 4.1 (ref. 36)) predicts a neutral titration state for H165 (Supplementary Fig. 10).

## Discussion

We have introduced a novel computational approach for characterizing complex structural transitions in membrane transporters, that allowed for atomic-level reconstruction of an entire thermodynamic cycle and its free energy profile in an MFS transporter. The wealth of detailed information provided by the performed simulations can be used to design several new experiments to test various aspects of the transport cycle and even trap and structurally characterize the putative outward-facing and/or intermediate states of GlpT.

For instance, one may design site-directed cross-linking experiments to trap the structurally unknown OF state, based on our finding that helices H5 and H11 undergo significant conformational changes during the IF→OF transition and are among the primary elements contributing to the occlusion of the cytoplasmic gate. In particular, using a 8-Å C^*α*^–C^*α*^ distance as an approximate measure for cross-link formation we have identified several residue pairs that are distant in the crystal structure (IF state) but form contacts in the OF state. Some of the specific residue pairs located on the cytoplasmic half of helices H5 and H11 that form contacts in the OF state include V158–G382, V158–A385 and I157–G382, which are too far from each other in the IF state (C^*α*^–C^*α*^ distances of 14–16 Å in the crystal structure). In addition, G142–G386, V146–G382 and R143–G382, are among the residue pairs on helices H4 and H11 that also reduce their distances on transition to the OF state and can form cross-links in this state. In the OF conformation, helices H5 and H8 form new contacts as well, for example, between S159 and G310 whose C^*α*^ atoms are ∼12 Å apart in the crystal structure (IF state). A similar behaviour is also detected for several other residue pairs; however, some of these pairs also form contacts on substrate binding while the transporter is still in the IF conformation (for example, V158 and G369 on helices H5 and H10, respectively).

We note that our approach can be generalized to consider the involvement of other ligands in the transport cycle. In GlpT, for instance, G3P binding and unbinding events as well as the G3P-bound IF↔OF transition can be studied using a protocol similar to that used here for inorganic phosphate. This study and the powerful approach introduced open opportunities for the study of MFS and other membrane transporters in their full chemical detail using enhanced sampling techniques and state-of-the-art supercomputing.

## Methods

### Computational approach overview

Here we will provide an overview of our computational approach, define the collective variables used in the simulations and briefly describe the sampling protocols. A brief description of the alchemical free energy calculations, PHSM, orientation-based biasing and analysis techniques used in this study are also provided. In the end, the theoretical framework of our study is discussed. More details on the sampling protocols, alchemical free energy calculations, PHSM and data analysis are provided in Supplementary Notes 1–4, respectively.

Characterizing large-scale structural transitions of membrane transporters without compromising the full-atomic description of these systems and their environments poses a major challenge to computational techniques due to the prohibitively long timescales involved in the process. Recognizing this issue, we have recently developed an efficient computational protocol towards describing large-scale conformational transitions using a combination of several distinct enhanced sampling techniques, some of which are discussed in our recent work on a primary transporter^19^,^20^.

Ideally, MD simulations can be used to characterize thermodynamic and kinetic properties of biomolecular processes, given adequate sampling. However, conventional integration techniques and sampling tools such as regular MD are far from ideal for the study of complex biomolecular systems and processes due to the large number of d.f. involved. Biased MD simulations provide a more practical alternative to brute-force MD for thermodynamic characterization of such complex systems. Thus, a time-dependent or time-independent biasing potential is used along with a *post-hoc* reweighting scheme to recover the unbiased statistics. Designing such biasing protocols, however, is quite challenging, given the dimensionality and complexity of the phase space in biomolecular systems. One particular simplification which makes biased MD relevant for the study of structural transitions is the premise of the existence of a low-dimensional manifold on which lie most of the relevant conformations visited by a system during a transition^37^,^38^ (see Theoretical framework section). Many enhanced sampling methods such as those used in the present study rely on this assumption that is mostly based on the high cooperativity of structural elements in biomolecules and their concerted motions. However, such a manifold or reaction coordinate is not known *a priori*, and poor choice of a reaction coordinate for biasing could result in irrelevant transition pathways and misleading free energy profiles. The novel sampling strategy presented in this work is designed specifically to address this problem.

The majority of the simulations performed here were based on the BEUS scheme^21^,^19^,^20^, a umbrella sampling^39^ method combined with Hamiltonian replica exchange^21^. In this scheme, biases are occasionally exchanged between the neighbouring windows (umbrellas), thereby increasing the chance of continuous sampling in the orthogonal d.f. that is necessary for a reliable free energy estimate. Despite its powerful sampling capability, BEUS also relies on the relevance of reaction coordinate it uses. For complex conformational free energy landscapes, an iterative sampling approach seems necessary in which the results of each iteration are used to design better reaction coordinates, to generate better initial conformations, and to better sample the relevant regions of the configuration space^20^,^40^. String method with SMwST^22^ or other path-finding algorithms can be used in parametrization of narrow pathways defined in high-dimensional spaces to be sampled robustly in following BEUS simulations. We have implemented a parallel version of the SMwST method in which each image consists of multiple parallel copies, restrained and released iteratively with periodic exchange of information between the replicas to update the image centres at the end of each iteration. A similar but independent implementation has been previously reported elsewhere^41^. While SMwST is ideally assumed to converge to the most probable pathway, it heavily relies on the relevance of its initial pathway which is provided by other methods (for example, targeted MD^42^). An algorithm to extract the most relevant pathway from a prior data set is desired to provide an optimized starting point for computationally demanding SMwST simulations. To extract an approximate minimum free energy path from samples generated by prior BEUS simulations (or any other sampling protocol, given a reweighting scheme is available) we have developed a non-parametric analysis technique, namely PHSM which is reminiscent of the finite-temperature string method^23^. The main difference between PHSM and other string methods such as finite-temperature string and SMwST is that PHSM uses an existing set of samples to extract a transition pathway. The PHSM path extracted from BEUS simulations could be further relaxed using SMwST^22^. Once the SMwST string is converged one may perform an extensive one-dimensional (1D) BEUS simulation using the image centres as the window centres. We thus use terms image and window interchangeably in this paper. Also note that, for BEUS simulations, we use one copy/replica per window/image, while for SMwST we use multiple copies/replicas per image.

### Collective variables

Our approach to design optimal reaction coordinates and biasing protocols is based on an empirical search, which relies on our knowledge of the system under study to limit the conformational sampling to the relevant regions of the phase space while keeping the calculations reliable and accurate. We design several mechanistically relevant, system-specific reaction coordinates whose usefulness and applicability to induce the transition of interest are examined using qualitative assessments along with nonequilibrium work measurements^20^,^43^,^44^. The approach provides an empirical framework for optimizing the biasing protocols in a series of short simulations. By using advanced system-specific biasing protocols, we can significantly improve the effectiveness of the search protocol in sampling complex transition pathways^19^,^20^,^45^.

To induce the IF_*a*_→OF_*a*_ transition, we initially defined several collective variables based on RMSD or orientation quaternions of different helices, bundles of helices or their linear combinations. Eventually, our initial IF_*a*_→OF_*a*_ pathway was generated using orientation quaternions *Q*_1_ and *Q*_7_ defined for TM helices H1 and H7, respectively. The reference structure was the initial IF_a_* conformation (modelled based on the crystal structure of GlpT and equilibrated in membrane; see Sampling protocol section). In most other simulations including the final set, results of which are provided in the main paper, we use a set of 20 orientation quaternions (denoted as {*Q*} for brevity) including *Q*_*i*_ and *Q*_*i*_′,*i*=1,…5, 7,…,11 in which *Q*_*i*_ and *Q*_*i*_′ are orientation quaternions of *i*th TM helix with respect to our IF_*b*_ and OF_*b*_ models, selected from simulation sets 4 and 6, respectively (see Fig. 1c,d and Supplementary Table 1). For an ideal rigid-body conformational change, using two different reference structures would be redundant; however, some helices undergo bending and kinking motions, thereby making a single-reference quaternion a poor choice (see Supplementary Fig. 3 for an example of a kinking motion observed in the simulations). To track the position of the substrate with respect to the protein along the lumen, we define 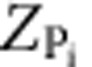 as 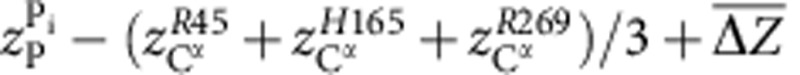, in which 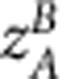 is the *z* position of atom *A* in residue *B*, and 
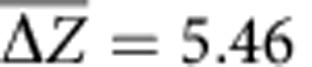
 is a constant. 
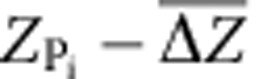
 is the *z* component of the vector connecting the center of mass of a select number of C^*α*^ atoms of protein (within the lumen) to the phosphorus atom of the P_i_. 
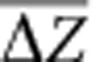
 does not play any role in the biasing potential since it is a constant; however, it was used to shift the results in all the plots such that 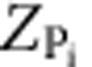 approximately represents the *z* position of the P_i_ with respect to the center of mass of the protein. Defining Δ*Z* as 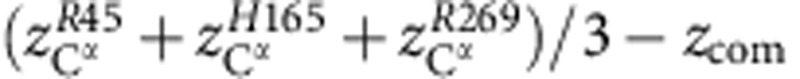, in which *z*_com_ is the *z* component of the center of mass of the C^*α*^ atoms of the protein, 
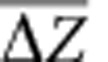
 can be estimated *a posteriori* as the mean value of Δ*Z* from all the sampled conformations with a s.d. of ∼0.56 Å. 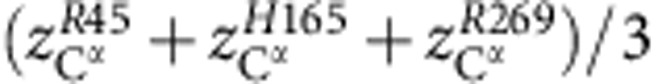 is thus relatively stable with respect to *z*_com_ and provides a numerically efficient alternative for the calculation of 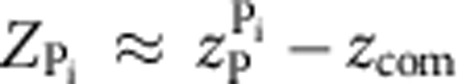, that is, the relative position of the substrate with respect to the protein projected onto *z*. The collective variable set ({*Q*},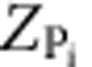) defines a coarse configuration space describing the conformation of the GlpT:P_i_ complex. In addition, in simulation set 10, we used an RMSD-based collective variable 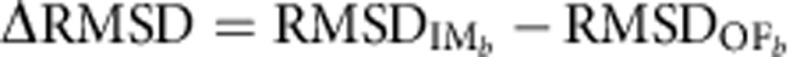 in which RMSDs are defined based on the C^*α*^ atoms of TM helical regions with respect to OF_*b*_ and IM_*b*_ conformations, selected from simulation sets 6 and 8, respectively (see Fig. 1c,d and Supplementary Table 1). Note that IM_*b*_ is a P_i_-bound conformation, representing a local minimum in simulation set 8 (see Supplementary Figs 11 and 12).

### Sampling protocol

In our previous studies^16^,^17^, we had prepared a membrane-embedded model of the *apo* GlpT in the IF state from the crystal structure of GlpT (PDB: 1pw4)^8^ and equilibrated the full-atomic model in an explicit POPE (1-palmytoil-2-oleoyl-*sn*-glycero-3-phosphatidylethanolamine) lipid bilayer and TIP3P (ref. 46) solvent environment. We used this equilibrated conformation of *apo* GlpT (denoted as 
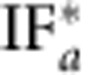
) as the initial conformation for the rest of the simulations which, for consistency, used similar simulation conditions to our previous simulations (see refs 16, 17 for details). The protonation states of all titratable residues in the crystal structure were assigned based on p*K*_a_ calculations using PROPKA^47^ and the protonation sites were determined using MolProbity^36^. The simulations were performed with NAMD 2.8–10 (ref. 48). A brief description of the protocols used in simulations listed in Fig. 1c,d and Supplementary Table 1 is given here, followed by a more detailed description of the parameters involved in the protocols such as the window centres and force constants of the BEUS simulations and the definition of metric in the SMwST and PHSM algorithms.

First we aimed at generating a reliable representation of the OF state. In brief, after trying several different biasing protocols, we identified the orientation quaternions of TM helices H1 and H7 (*Q*_1_,*Q*_7_) as a set of collective variables capable of inducing the IF→OF transition in *apo* GlpT. Imposing a rotational change on these helices in a direction perpendicular to the pseudosymmetry plane induces a global conformational change in the protein resulting in a locally stable conformation, which is open to the periplasm and closed to the cytoplasm (OF state). The resulting trajectory was used for an iterative optimization process using: (i) 1D BEUS along the nonequilibrium pathway defined in the (*Q*_1_,*Q*_7_) space (simulation set 1); (ii) SMwST using collective variable {*Q*} (simulation set 2); and (iii) 1D BEUS along the converged SMwST path defined in the {*Q*} space (simulation set 3). In the second stage we performed several sets of BEUS simulations based on equilibrated IF_*a*_ and OF_*a*_ states by adding the substrate (an inorganic divalent phosphate P_i_^−2^, denoted as P_i_ in this paper) to the system to identify IF_*b*_ and OF_*b*_ states. The BEUS simulations for both IF and OF states were performed using reaction coordinate 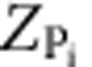 (simulation sets 4 and 6), followed by a second set of BEUS simulations in the ({*Q*},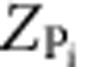) space initiated from PHSM-generated initial pathways (simulation sets 5 and 7). Finally, the most complex process (within the context of our computational approach) involved the IF_*b*_↔OF_*b*_ transition. The complication was particularly due to the fact that the large-scale conformational change of GlpT is accompanied by a ∼8-Å translocation of the substrate and a significant local conformational change within the biding site. To find the optimum pathway coupling the global conformational changes to substrate translocation within the binding site, we performed 1D BEUS simulations along (*Q*_1_,*Q*_7_) and 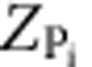 spaces, followed by a two-dimensional BEUS in (ΔRMSD, 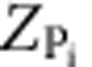) space (simulation sets 8, 9 and 10). A minimum free energy pathway was extracted from the generated data using PHSM which was further relaxed using SMwST simulations in the ({*Q*},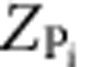) space (simulation set 11). The converged SMwST path was used to initiate a 1D BEUS simulation in the ({*Q*},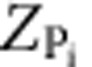) space (simulation set 12). The final set of simulations combined the most relaxed pathways discussed above (extracted using PHSM) to sample along the optimum cyclic pathway in the ({*Q*},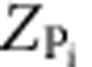) space connecting OF_*a*_, OF_*b*_, IF_*b*_ and IF_*a*_ states of the GlpT:P_i_ complex (simulation set 13). Note that in the final set (unlike other simulation sets), the *apo* GlpT is modelled by restraining the substrate outside the protein at a particular 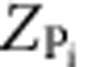 (that is, ∼−35 Å). P_i_ is also restrained in all simulations to stay within a cylinder of radius 15 Å (aligned with the membrane normal and centred in the lumen). The entropic gain of ‘free' P_i_ in the bulk is therefore ignored in our free energy calculations (Fig. 1e). In addition, the free energy differences are reported based on their values at extrema, which do not take into account the shape of the free energy landscape. A more detailed description of the parameters involved in these simulations is provided in Supplementary Notes 1.

### Nonequilibrium alchemical free energy calculations

We have calculated relative binding and conformational free energies associated with GlpT R45K mutation using bidirectional fast-growth thermodynamic integration (FGTI)^32^,^33^ on four major simulated states of GlpT:Pi complex including OF_*a*_, IF_*a*_, OF_*b*_ and IF_*b*_ (Supplementary Fig. 9). First we equilibrated the four representative systems associated with these minima generated from simulation set 13 and extracted using the PHSM algorithm, each for 100 ns (Supplementary Fig. 1f–i). The last 50 ns of these unbiased equilibrium simulations were used to initiate the forward wild type (WT) to R45K transformations. In all, 250 initial conformations (collected every 200 ps) were selected to build dual-topology models for R45K mutation using VMD (ref. 49) plugin Mutator (version 1.3). Each model was then minimized for 1,000 steps and equilibrated for 20 ps before a 100-ps-long FGTI simulation performed using FEP module in NAMD 2.10 (ref. 48). A scaled-shifted soft-core potential^50^ was used for van der Waals interactions, electrostatic interactions were scaled for outgoing and incoming atoms in two non-overlapping stages, and only nonbonded interactions of perturbed atoms were scaled with their environment.

Since the coupling parameter λ was varied at every step, all forces were also calculated at every step; however, the rest of the simulation parameters were the same as those used for other MD simulations in this study (see Sampling protocol section for details). For the reverse R45K to WT (or K45R, for brevity) transformations, the snapshots at *t*=50 ns from unbiased equilibrium simulations shown in Supplementary Fig. 1f–i were used to build a R45K mutant, which was then minimized for 1,000 steps and equilibrated for 5 ns before a 50-ns production run to generate 250 conformations which were then minimized, equilibrated and transformed using FGTI scheme similar to the forward simulations. For each FGTI simulation, the nonequilibrium work, *W* was calculated as a sum over 
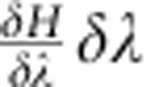
 values reported at every step. The free energy difference due to R45K mutation was estimated for each state using forward and reverse work distributions and employing both Crooks–Gaussian intersection^51^ and Bennett acceptance ratio^52^,^53^ methods. A replacement-based bootstrapping technique was used for the error analysis in Crooks–Gaussian intersection method while an analytic expression was employed for estimating the error in Bennett acceptance ratio method^53^. See Supplementary Note 2 for a discussion on how relative binding and conformational free energies were estimated based on relative free energies of individual states between WT protein and R45K mutants.

### *Post-hoc* string method

Here we introduce PHSM, an analysis method to extract, from an existing sample set, a principal curve defined in a multidimensional collective variable space **ζ**, whose intersection with any perpendicular hyperplane coincides with the Boltzmann-averaged center of that hyperplane. We assume that a set of samples {**ζ**^*t*^} are generated from a single or multiple BEUS simulations, representing a continuous region of the **ζ** space. The weights {*w*^*t*^} associated with samples are assumed to approximately represent the Boltzmann distribution in the sampled region. A metric is defined in the **ζ** space to measure the distance/closeness between conformations. The choice of **ζ** space (and its metric) is based on our analysis of the sampled data to include all slow d.f. including (i) those used in prior BEUS simulations and (ii) those detected as undersampled, slow d.f. not included in those simulations. The metric provides a way of prioritizing different d.f. in ensuring their continuity in the converged pathway. We use an algorithm similar to ‘k-means' clustering to find the optimum centroidal Voronoi tessellation^54^ along a pathway connecting two given points in the **ζ** space in a non-parametric manner. We start with an initial pathway that is represented by a string of *N* images with centres {**ζ**_*i*_} (iteration 0). At each iteration, these centres are updated according to the following algorithm: (i) Voronoi cell *B*_*i*_ is built for each image which is a set of all samples being closer to **ζ**_*i*_ than any other image center:


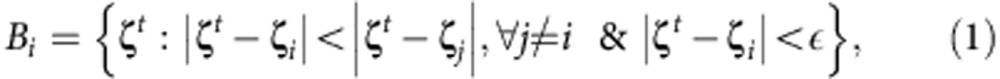


in which 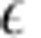 is a parameter which determines the thickness (or localization) of the transition tube; (ii) the Boltzmann-averaged center of each cell 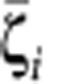 is determined by averaging over **ζ**^*t*^ of all *B*_*i*_ samples with their appropriate weights; and (iii) the averaged centres are used to parametrize a Bézier curve 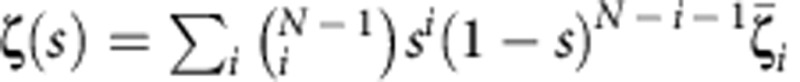, and *N* equidistant points are specified along the curve between **ζ**(0) and **ζ**(1) to be used as new image centres {**ζ**_*i*_} for the next iteration. Steps (i–iii) will be repeated until the image centres are converged to a string representing the principal curve that is an approximate minimum free energy path. The closest sampled conformation to each image center may be used to reconstruct a trajectory to be used as an initial pathway for follow-up BEUS or SMwST simulations (see Supplementary Fig. 13 for examples of the application of the PHSM algorithm). Particular parameters used in our PHSM analysis are provided in Supplementary Note 1 while a more detailed description of PHSM algorithm is provided in Supplementary Note 3.

### Orientation-based biasing

One particular feature that seems to best describe a variety of large-scale conformational changes is semi-rigid-body orientation change of protein structural elements or domains. The orientation quaternion technique^55^,^56^,^57^ has proven successful as a practical method for defining well-behaved collective variables, specifically aimed at inducing inter-domain orientation changes^19^,^20^. Quaternion *Q*=(*q*_0_, *q*_1_, *q*_2_, *q*_3_) can be considered as a composite of a scalar *q*_0_ and an ordinary vector **q**=(*q*_1_, *q*_2_, *q*_3_), or as a complex number *Q*=*q*_0_+*q*_1_*i*+*q*_2_*j*+*q*_3_*k* with three different imaginary parts. The optimal rotation to superimpose one set of coordinates on another can be described by a unit quaternion, that is, orientation quaternion 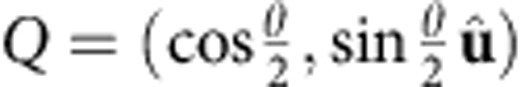 in which *θ* and 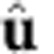 (a unit vector) are the optimum angle and axis of rotation, respectively.

As a collective variable, an orientation quaternion can be used to induce a rotational change on a given domain (for example, a helix or a bundle of helices) or simply restrain its orientation by using the harmonic potential:


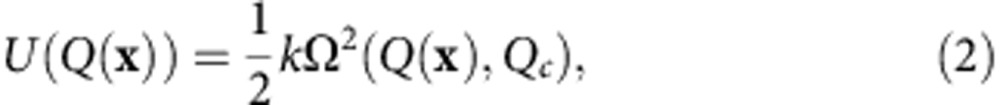


in which *Q*(**x**) is the orientation quaternion of a set of *N* atoms (**x**={**x**_*i*_: *i*=1,…,*N*}) with respect to a reference set, and *Q*_*c*_, the center of the harmonic potential, could be time-independent (for restraining) or time-dependent (for inducing a transition). Ω(*P*,*Q*) is the length of the geodesic between two points on the unit sphere, transformed by orientation quaternions *P* and *Q* from an arbitrary point on the same sphere. An approximate estimate for Ω(*P*,*Q*) (which is used in NAMD/LAMPS implementation^57^) is arccos(|*P*·*Q*|) in which *P*·*Q* is the inner product of *P* and *Q*. Note that for small Ω, that is, small deviation from the harmonic center—which is relevant in the stiff-spring approximation—one can show Ω(*P*,*Q*)^2^≈|*P*−*Q*|^2^ thus relation (2) reduces to 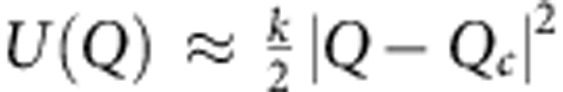.

### Analysis techniques

The configurations were collected every 5 ps from MD trajectories and analysed using VMD^49^, ProDy^58^, HOLE^59^, PROPKA^34^,^35^,^47^ and MolProbity^36^ along with a number of in-house C++ and Python codes. By discarding the first 10 ns of each replica in simulation set 1 (see Supplementary Note 4 for justification), an ensemble of 1,200,000 configurations (150 replicas × 40 ns/5 ps) was generated and analysed, results of which are shown in Figs 1, 2, 3, 4 and Supplementary Figs 2–8 and 10 as well as Supplementary Tables 2–3. To estimate the error associated with the free energies, a Bayesian block bootstrapping algorithm^20^ was used with a block size of 10 ns (see Supplementary Note 4 for justification), resulting in 600 blocks (150 replicas × 40 ns/10 ns). The stiff-spring correction term of free energies in Fig. 1d was estimated to be smaller than 0.3 kcal mol^−1^ for all points, which is also smaller than the statistical error estimated from the bootstrapping algorithm (see Theoretical framework). In our analysis of the final simulation set, an unweighted estimator was used to track the average behaviour of the transition pathway projected onto different spaces with reported errors taking into account the statistical inefficiency of the correlated data points. Statistical inefficiencies were estimated based on the autocorrelation times such as those reported in Supplementary Table 2. Note that the autocorrelation times are calculated based on the continuous trajectories associated with replicas rather than reconstructed trajectories based on images, thereby providing an upper bound for the actual autocorrelation times of images.

### Theoretical framework

Suppose that the dynamics of a high-dimensional atomic system {**x**} can be simplified as an effective dynamics in a coarse variable space **ζ**. The effective dynamics can be described by a Brownian motion in the **ζ** space with an effective potential energy *G*(**ζ**) and diffusion tensor **D**(**ζ**). The former is the PMF of the atomic system in the **ζ** space and the latter is generally position-dependent and anisotropic^60^. One may sample the regions around a given point **η** in the **ζ** space by adding a biasing term to the potential of the atomic system such as 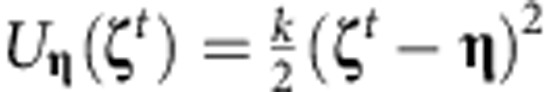 in which **ζ**^*t*^ is the instantaneous value of collective variable **ζ** at time *t* and *k* is the force constant. The free energy of the biased system (or the perturbed free energy) *F*(**η**) is:


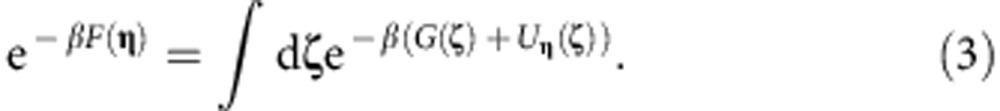


Generalizing the formula in ref. 61, one can show that the perturbed free energy at *F*(**ζ**) and the PMF *G*(**ζ**) are related via:


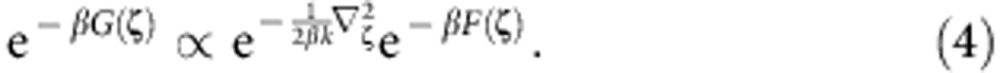


For large *k*, that is, in the stiff-spring approximation^62^, one may expand the above relation to extract the first two terms in 1/*k* (ref. 61):


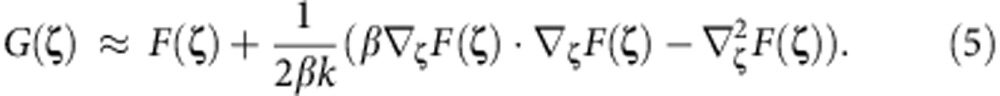


Thus, for large force constants, the PMF can be approximated using the perturbed free energy *F*(**ζ**). The validity of this approximation can be tested by *a posteriori* comparison of the two terms, assuming the gradient and Laplacian of the perturbed free energy are estimated as well—which is numerically challenging in a high-dimensional space.

Ideally, one may use a 1D collective variable for defining the effective dynamics as well as the biasing protocol. In practice, however, this may only be possible for extremely simple systems. A practical solution to this problem is to keep the collective variable space multidimensional, while sampling only around a particular pathway, represented by a 1D curve **ζ**(*s*), parametrized by *s*. The choice of the pathway is obviously crucial here and determines the relevance of the free energy results to the transition of interest. Several path-finding algorithms have been proposed which iteratively/adaptively refine an initial pathway to converge to a final pathway satisfying a given criterion, for example, by minimizing the free energy or maximizing the flux^22^,^23^,^63^,^64^,^65^. Among them is SMwST^22^ that is used in this study as well. We have also developed a novel algorithm, that is, PHSM, for extracting pathways from the results of prior simulations to initiate other simulations, a well-suited algorithm for iterative simulations in which a good set of reaction coordinates are not known *a priori*.

Assuming **ζ**(*s*) approximately represents the minimum free energy path, and *s* is its arc length, relation (5) can be simplified to:


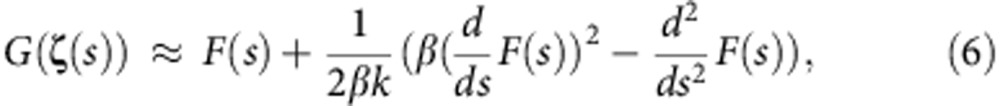


in which *F*(*s*) is, up to an additive constant, the free energy associated with the system perturbed by biasing potential 
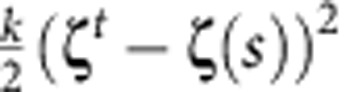
, and 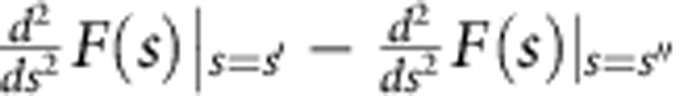 is assumed to dominate 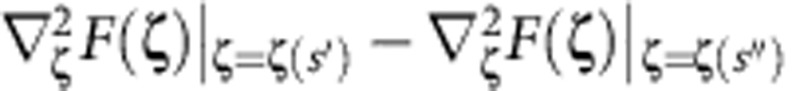. Under this assumption, the validation of stiff-spring approximation requires the evaluation of *F*(*s*) and its first and second derivatives with respect to the arc length *s*. To numerically estimate *F*(*s*), one may use umbrella sampling^39^ to discretize *s* and define *N* umbrella windows/images with biasing potentials 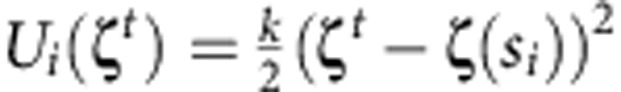 for *i*=0,…,*N*−1. This scheme can be thought of as a 1D umbrella sampling along the reaction coordinate *s* with an additional restraint on the (shortest) distance from the **ζ**(*s*) curve. Perturbed free energies *F*_*i*_=*F*(**ζ**(*s*_*i*_)) can be estimated (up to an additive constant) by self-consistently solving the equations^66^,^67^:


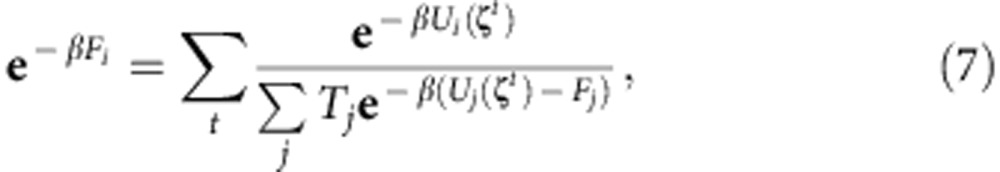


in which ∑_*t*_ sums over all collected samples (irrespective of which replica or image they belong to) and *T*_*j*_ is the number of samples collected for image *j*.

With appropriate reweighting, PMF can be reconstructed in any arbitrary collective variable space, given sufficient sampling in that space. *w*^*t*^, the unnormalized weight of configuration **x**^*t*^ can be estimated via ref. 66:


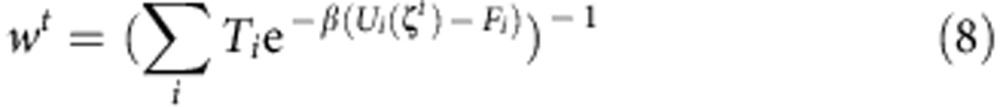


in which {*F*_*i*_} are estimated via equation (7). Alternatively^66^, one may estimate {*w*^*t*^} and {*F*_*i*_} by iteratively solving equation (8) and:


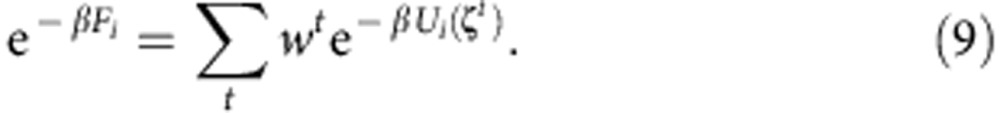


The PMF in terms of **ξ**(**x**), an arbitrary collective variable, is estimated (up to an additive constant) as:


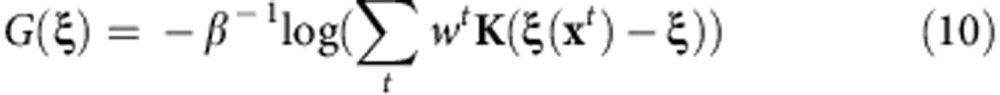


in which **K** is a kernel function. The above estimator is not accurate if the sampling in **ξ**(**x**) is not converged which is the case if **ξ**(**x**) has a slow dynamics and is not strongly correlated with **ζ**. For the special case of **ξ**=**ζ**, the perturbed free energies {*F*_*i*_} can be used directly to estimate the PMF in the stiff-spring approximation.

The reweighting schemes discussed so far are based on maximum-likelihood (or maximum *a posteriori*) estimation, while in this study we have used a Bayesian estimation method to avoid overfitting^68^. One may use a Gibbs sampler to draw {*w*^*t*^} and {*f*_*i*_} (*f*_*i*_ :=*T*_*i*_ exp(*βF*_*i*_)) from:


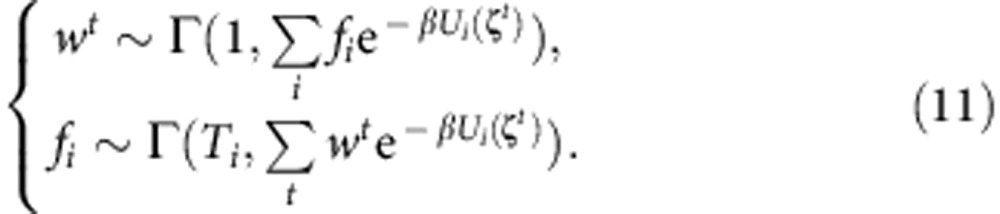


in which {*w*^*t*^} is normalized after each iteration and *x*∼Γ(*a*,*b*) indicates that *x* is drawn from a Gamma distribution of shape *a* and rate *b* (that is, *p*(*x*;*a*,*b*)∝*x*^*a*−1^exp(−*bx*)). On convergence, any ({*w*^*t*^},{*F*_*i*_=*log*(*f*_*i*_/*T*_*i*_)/*β*}) drawn is considered equally likely and can be used for free energy or PMF reconstruction. The mean and its standard error can be used to estimate any quantity of interest and its associated error. However, the error will be underestimated if the samples are correlated. A Bayesian block bootstrapping technique can be used to efficiently estimate the error associated with such correlated data points as already discussed elsewhere^20^,^69^.

The reweighting scheme described above is general for any arbitrary set of biasing potentials; however, to approximate *G*(**ζ**(*s*_*i*_)) by {*F*_*i*_} and to examine the stiff-spring approximation by evaluation of the second term of the expansion in relation (6), and more importantly to relate *G*(**ζ**(*s*_*i*_)) to the kinetics even qualitatively, one needs to make an assumption that **ζ**(*s*_*i*_) is an approximation of the minimum free energy path. Assuming the above (maximum-likelihood or Bayesian) estimators result in a smooth function for *F*(*s*), the first and second derivatives can be numerically estimated via finite difference methods from {*F*_*i*_} to estimate the second stiff-spring approximation terms.

Finally, for averaging an arbitrary quantity *A*(**x**) along the pathway **ζ**(*s*), one may use the weighted average 
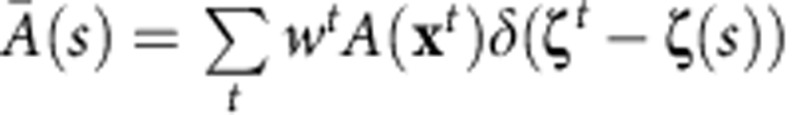
. However, in the stiff-spring approximation, unweighted estimator 
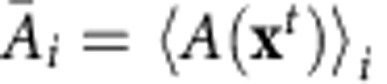
 is often more efficient. 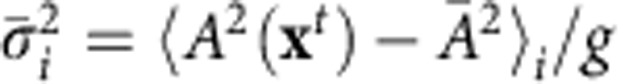 provides an estimate for the variance, given 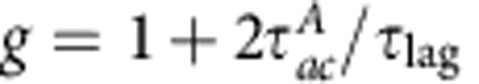 is the statistical inefficiency in which 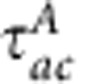 is the autocorrelation time associated with quantity *A* and *τ*_lag_ is the lag time between the data points used in the analysis^70^.

## Additional information

**How to cite this article**: Moradi, M. *et al*. Atomic-level characterization of transport cycle thermodynamics in the glycerol-3-phosphate:phosphate antiporter. *Nat. Commun.* 6:8393 doi: 10.1038/ncomms9393 (2015).

## Supplementary Material

Supplementary Information Supplementary Figures 1-13, Supplementary Tables 1-3, Supplementary Notes 1-4 and Supplementary References

Supplementary Movie 1 IF-to-OF conformational transition of apo and Pi-bound GlpT, along with phosphate binding/unbinding in the IF and OF states, representing a full thermodynamic cycle. This trajectory is reconstructed using the post-hoc string method (PHSM) based on our final bias-exchange umbrella sampling (BEUS) simulation set (Set 13). The free energies estimated along the 150 images (representing this cyclic transition pathway) reveal that substrate binding lowers the free energy barrier of the IF-to-OF transition. The ensemble-averaged pore radius for each image was calculated with HOLE (Smart et al., J. Mol. Graphics, 14, 354-360 (1996)) using all protein and phosphate atoms.
