# Supplementary material for: Atomic-level characterization of transport cycle thermodynamics in the glycerol-3-phosphate:phosphate antiporter
Source: Nat Commun. 2015 Sep 29;6:8393. doi: 10.1038/ncomms9393 (PMC4598623; doi:10.1038/ncomms9393)
Supplement: Supplementary Information — Supplementary Figures 1-13, Supplementary Tables 1-3, Supplementary Notes 1-4 and Supplementary References [file ncomms9393-s1.pdf]

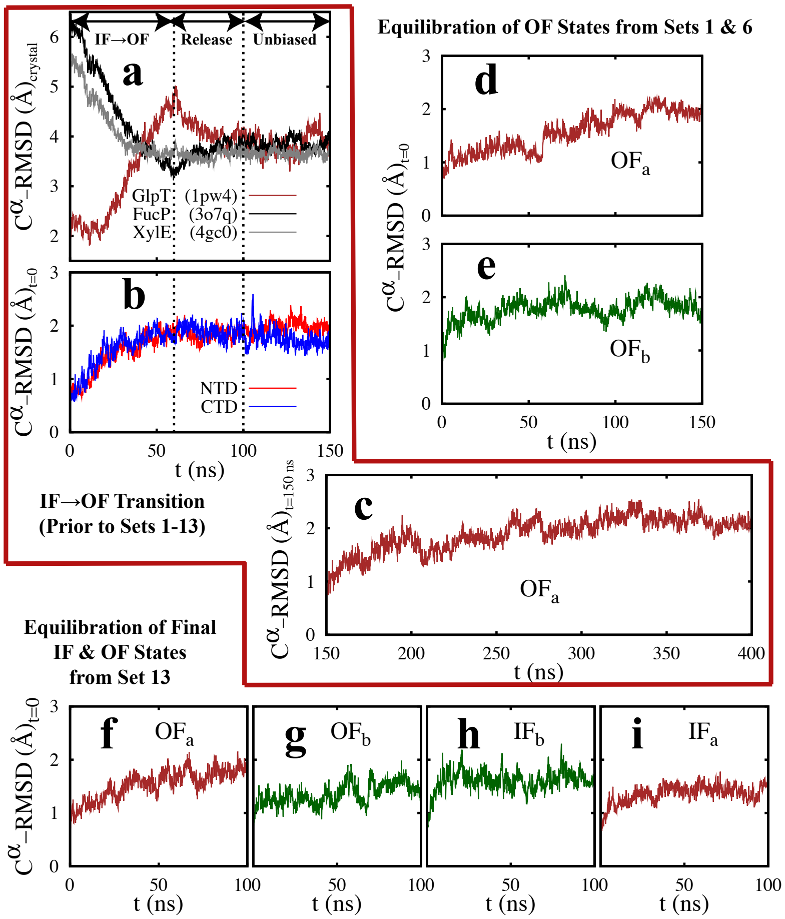

Supplementary Figure 1: **Generating and equilibrating the OF state of GlpT. a,b** Initial stage of generating GlpT OF_a state using a 60-ns long nonequilibrium simulation (based on (Q_1, Q_7) collective variables), followed by gradual release of the restraint (from t =60 to 100 ns) and unbiased MD (from t =100 to 150 ns) (**a**,**b**). Panel **a** shows the C^α RMSD of helical regions of the protein relative to the crystal structures of GlpT (brown), FucP (black), and XylE (gray). Panel **b** shows the internal C^α RMSD of helical regions of the NTD and CTD domains with respect to the initial conformation (*i.e.*, IF*). While the initial conformations of Set 1 (Fig. 1c) were selected from the simulation whose RMSD analysis is shown in panels **a,b**, we further equilibrated the generated OF_a state; Panel **c** shows C^α RMSD of helical regions of the protein relative to the snapshot at t = 150 ns. **d,e** Equilibration of OF_a and OF_b states generated using Simulation Sets 1 and 6, respectively. **f,i** Equilibration of final OF_a, OF_b, IF_b and IF_a states extracted from Simulation Set 13 (using PHSM algorithm).

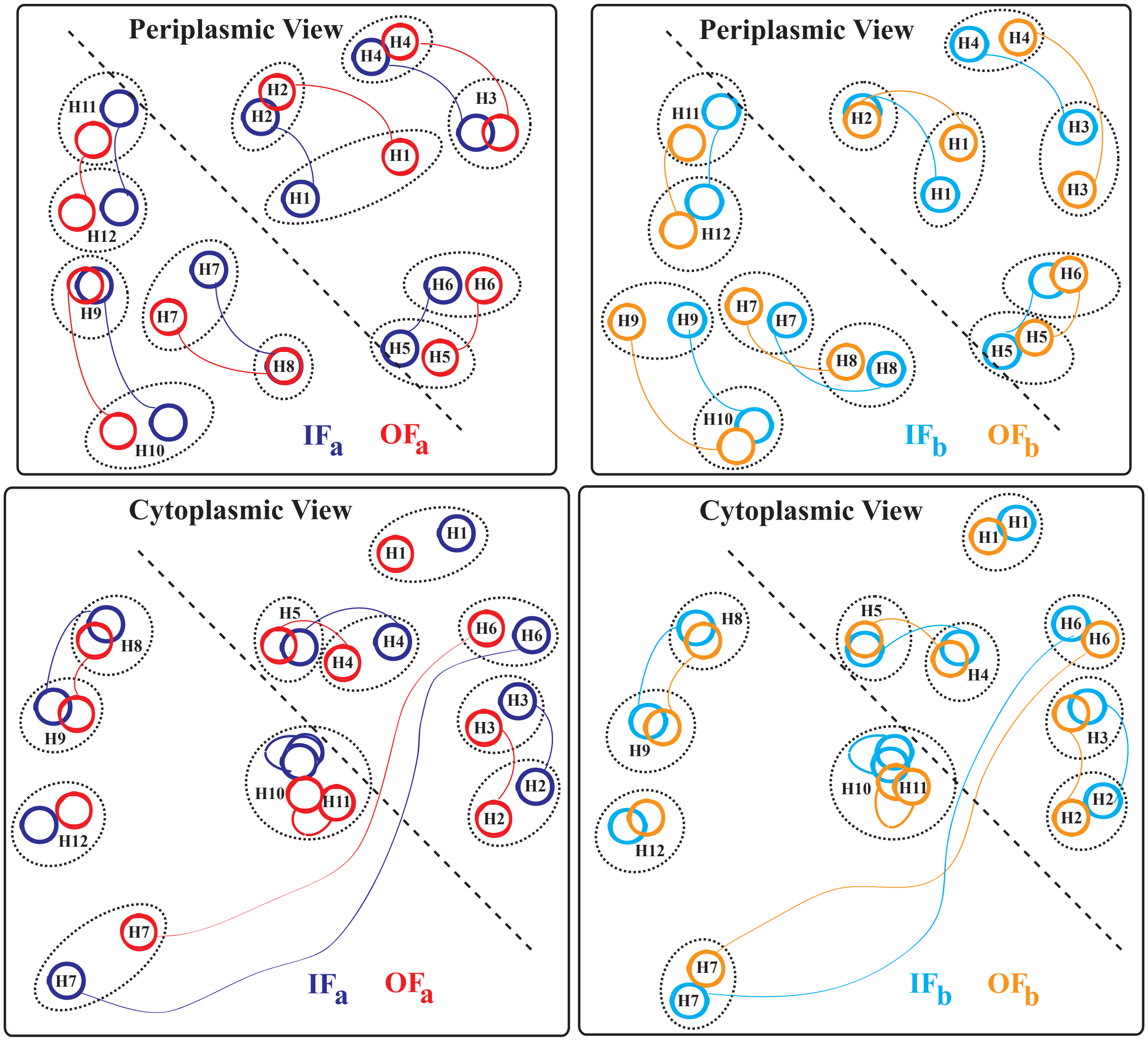

Supplementary Figure 2: **Illustration of rotational changes in TM helices using a simplified representation.** Each helix is represented by a straight rod (built using the VMD plugin Bendix [1]) whose top and bottom surfaces are shown from the peri- (top panels) and cytoplasmic (bottom panels) sides, respectively. Solid curves, schematically, represent the peri- (top panels) and cytoplasmic (bottom panels) loops, respectively while dashed lines approximately represent the pseudosymmetry plane. The conformational dynamics of GlpT involves internal conformational changes of helices, *e.g.*, bending, which is not captured in these simplified representations.

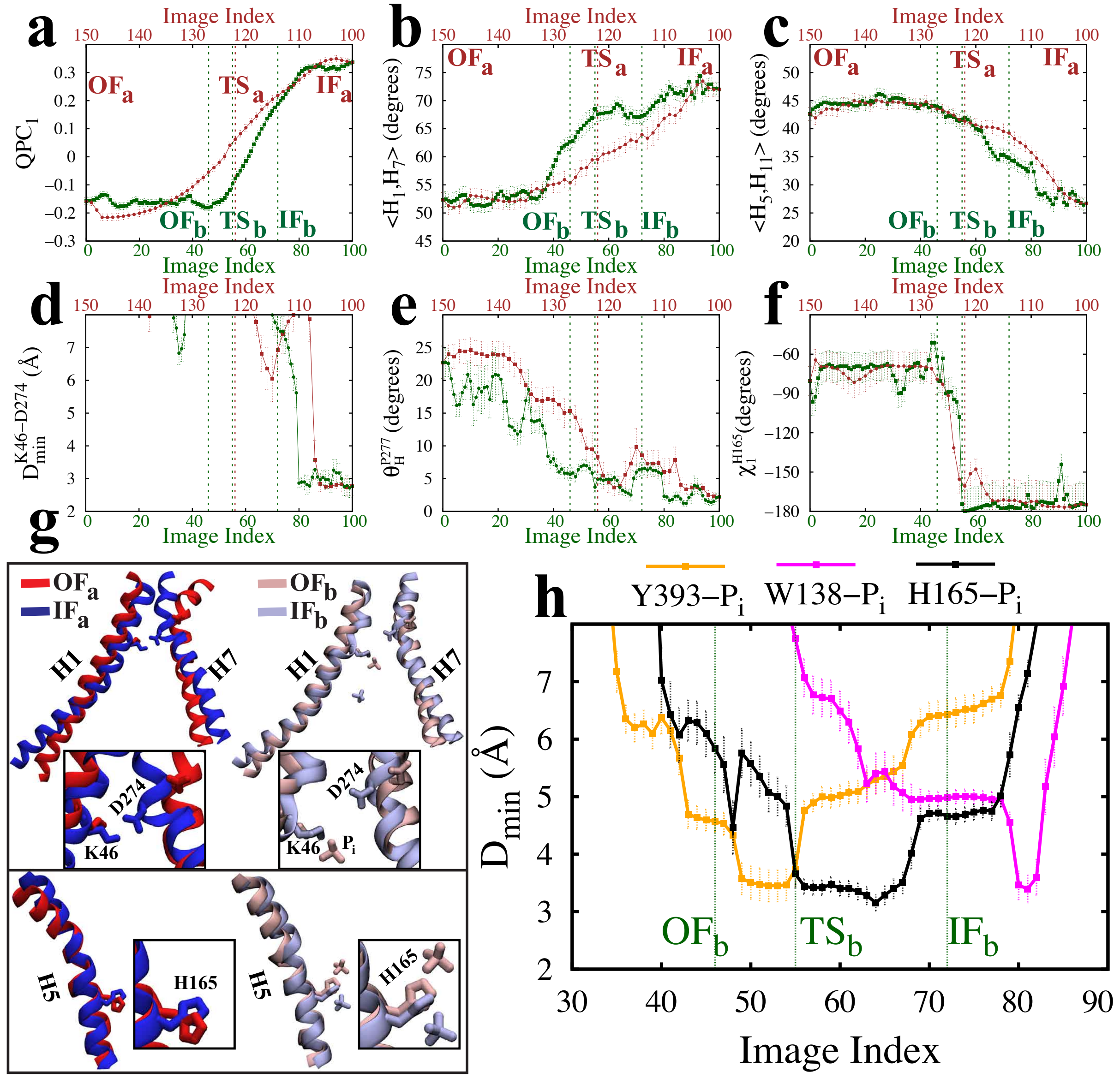

Supplementary Figure 3: **Coupling between global and local conformational changes. a** QPC_1, **b** <H1,H7>, **c** <H5,H11>, **d** D_{min} between K46 and D274 side chains, **e** local helical bending angle [2] at P277 on TM helix H7 (defined based on the C$^\alpha$ atoms of residues 274–277), and **f** χ_1 side-chain dihedral angle of residue H165 on TM helix H5, averaged over all conformations sampled for each image. **g** Representative conformations of TM helices H1/H7 (top) and H5 (bottom) in OF$_a$ (red), IF$_a$ (blue), OF$_b$ (light red), and IF$_b$ (light blue) conformations. Salt-bridge forming residues K46 and D274 (top), residue H165 (bottom), and P$_i$ (top and bottom) are shown in licorice representation. **h** D_{min} of phosphate and a select number of residues in the lumen (with a minimum D_{min} (among all images) between 3 and 3.5 Å). See Fig. 4 for the definition of D_{min}. The error bars represent the standard deviation (see Analysis techniques in Methods).

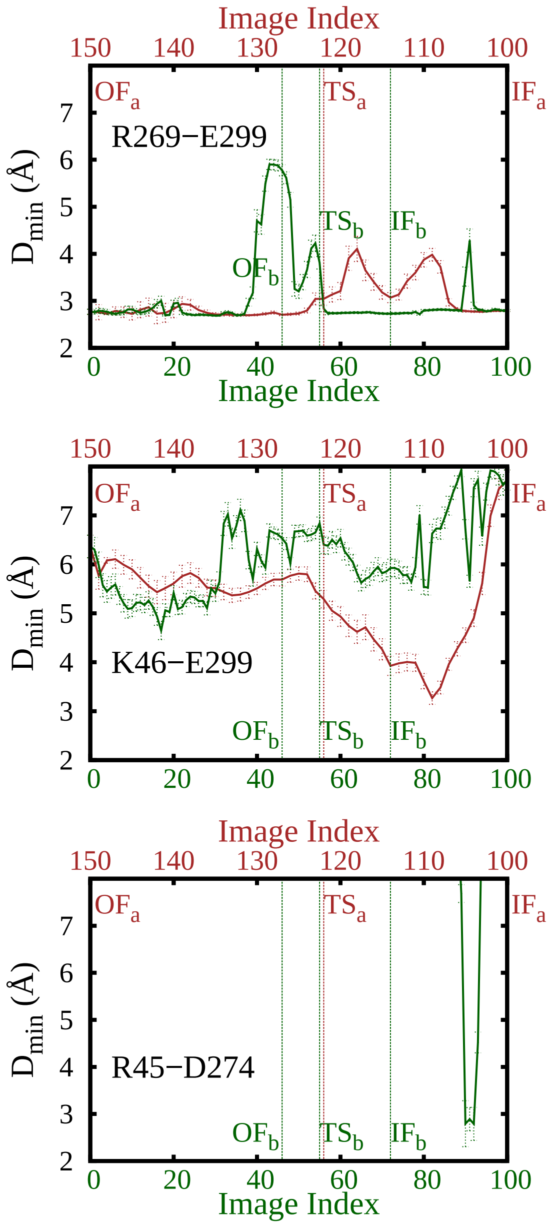

Supplementary Figure 4: **Salt bridge dynamics within the lumen.** D_{min} of the salt-bridge forming residues within the lumen (excluding that of K46-D274 which is shown in Supplementary Fig. 3). R269-E299 salt bridge is present in most of the images except around the OF$_b$ state in which R269 is involved in substrate binding (Fig. 4f). In the two other cases, salt-bridge formation is limited to a few images. K46-E299 and R45-D274 salt-bridges form only briefly (images 108–110, and 91–93, respectively) while the protein conformation is close to IF$_a$. The error bars represent the standard deviation (see Analysis techniques in Methods).

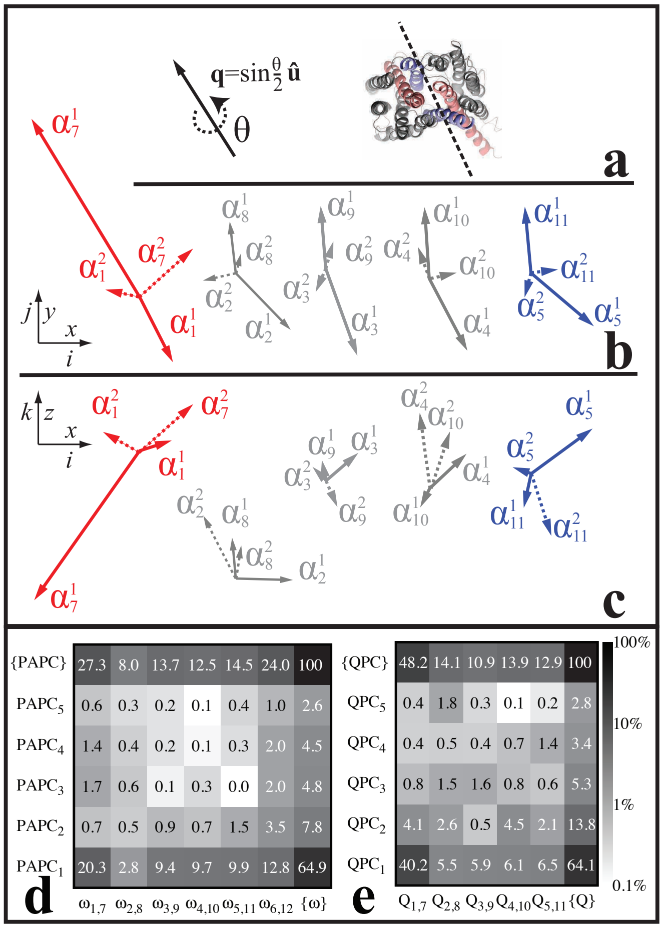

Supplementary Figure 5: **Dimensionality reduction. a–c** Geometric illustration of dominant quaternion principal components. Here $QPC_l = \sum_i(\mathbf{a}_i^l \cdot \mathbf{q}_i + \mathbf{a}_i^{\prime l} \cdot \mathbf{q}_i^\prime)$ and $\alpha_i^l = \sqrt{\lambda_l}\mathbf{a}_i^l$ in which $\mathbf{q}_i$ is the vector part of Q_i (see Methods), $\mathbf{a}_l^i$ is the coefficient of this vector in the lth QPC eigenvector, and λ_l is the lth QPC eigenvalue (or the variance of QPC_l based on all sampled conformations). The direction of α_i^l indicates the axis of rotation while its magnitude indicates the amount of rotational change relative to the other helices/QPCs. **a** Periplasmic view of GlpT structure (right) and a visual illustration of the vector part ($\mathbf{q}$) of an orientation quaternion, in which $\hat{\mathbf{u}}$ and θ are the axis and angle of rotation. **b** Projection of α_i^l onto the xy (or ij) plane for $l = 1, 2$. **c** Projection of α_i^l onto the xz (or ik) plane for $l = 1, 2$. Note that membrane normal is along the z (or k) axis. It is clear that TM helix H7 contributes the most to both QPC1 and QPC2. **d,e** Fraction of variance explained by different **d** principal-axis principal components (PAPC) and **e** quaternion principal components (QPC), for different TM helices. $Q_{l,m}$ denotes the orientation quaternions associated with helices l and m while $\omega_{l,m}$ denotes the roll axes of these helices (*i.e.*, the third principal axis component of each helix). $\{Q\}$ and $\{\omega\}$ denote the quaternions and roll axes of all helices (except for H6 and H12 in the former case), respectively. $\{QPC\}$ and $\{PAPC\}$ denote all quaternion and principal-axis principal components, respectively.

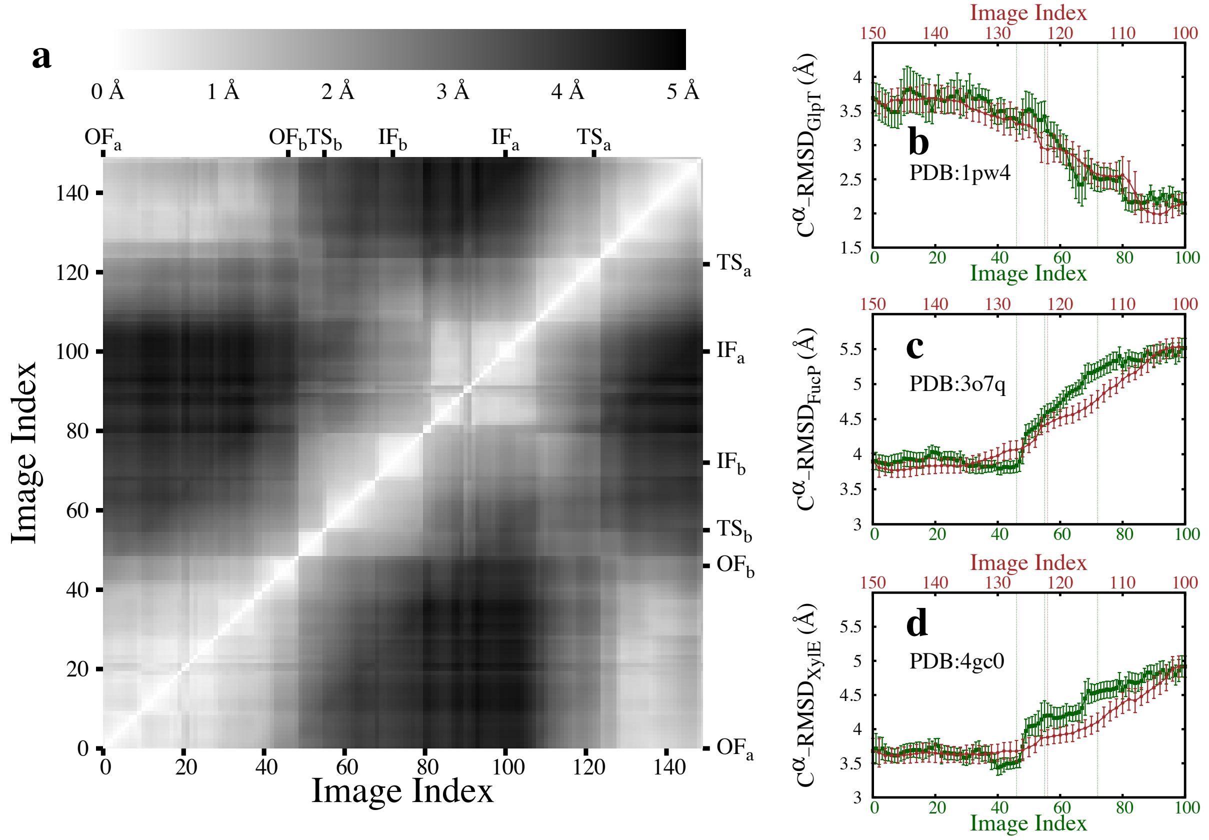

Supplementary Figure 6: **RMSD-based analysis of 150 sampled images.** **a** Pairwise C^{α}-RMSD of all 150 images. The first 300 C^{α} principal components (PCs) of all sampled conformations, which explain ∼99.00% of the variance, were used to estimate the RMSDs. The mean value and standard deviation of each PC of each image was estimated, and the pairwise RMSDs and their associated standard deviations were calculated. The standard deviations of pairwise RMSDs are between 0.4 and 0.8 Å. Pairwise RMSDs and their standard deviations are listed in Supplementary Table 3 for a select number of images. **b–d** Comparison between different sampled GlpT conformations and a select number of MFS crystal structures. TM helical region C^{α}-RMSD of 150 images (averaged over all sampled conformations for each image) with respect to crystal structures of GlpT [3], FucP [4], and XylE [5] which are in IF$_a$, OF$_a$, and OF$_b$ conformations, respectively. 306, 292, and 284 residues were used in the definition of RMSDs, for GlpT, FucP, and XylE reference structures, respectively. For FucP and XylE the selection of residues was based on structural alignment of the entire protein (in the OF$_a$ conformation) (using VMD's MultiSeq [6]); however, only the TM helical regions were used in the calculation of RMSDs. The error bars represent the standard deviation (see Analysis techniques in Methods).

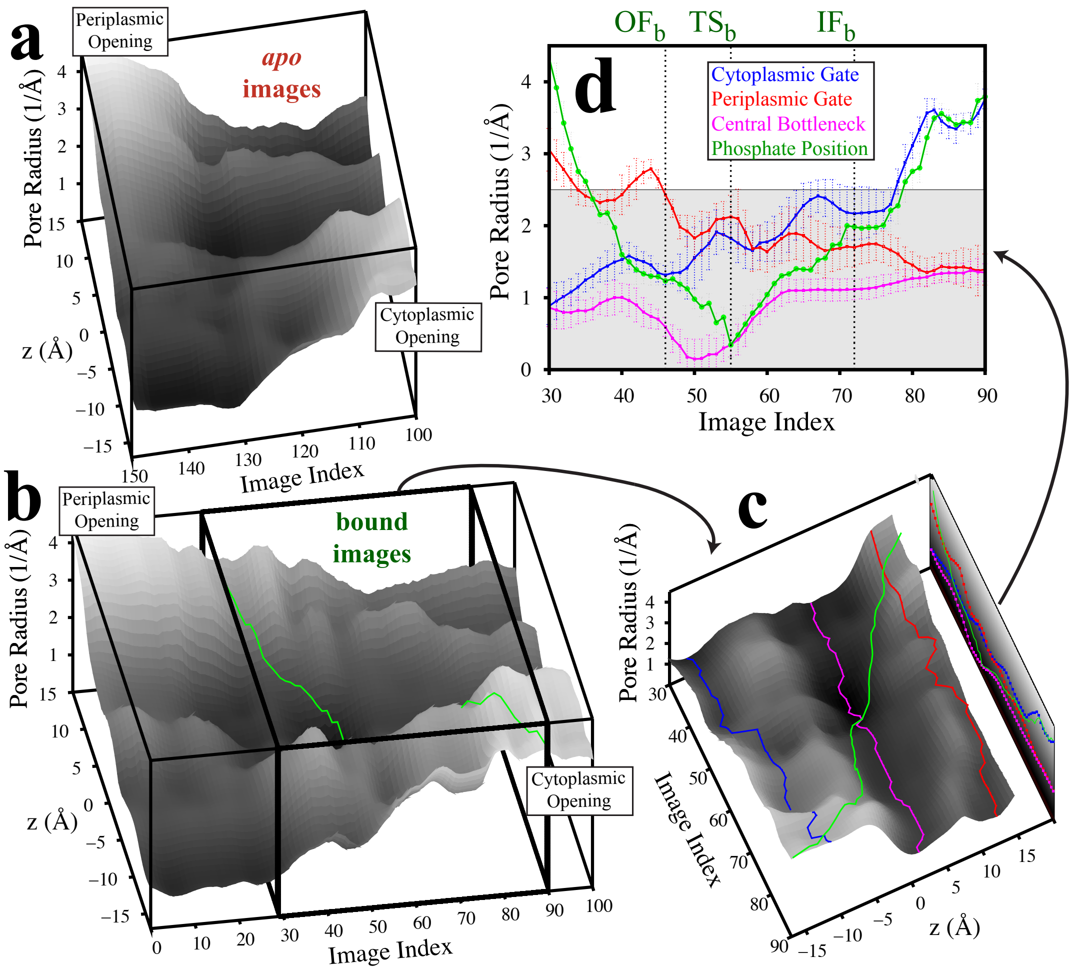

Supplementary Figure 7: **Lumen pore radius analysis. a,b** Pore radius at different z positions along 150 simulated images of GlpT, averaged over sampled conformations of each image (with no reweighting). The pore radius along the z axis (membrane normal) for each conformation was measured with HOLE [7] using all protein and phosphate atoms. Green line in **b** shows the position of the substrate along the z axis. **c** Same as **b** for images 30–90. Red, magenta, and blue lines represent the pore radius and its z position at periplasmic gate, central bottleneck, and cytoplasmic gate which are defined, at each image, as the minimum pore radius in $z \in [-20, -10]$, $z \in [-5, 5]$, and $z \in [10, 20]$, respectively (z values are in Å). The most frequently involved residues in the periplasmic gate, central bottleneck, and cytoplasmic gate determined from HOLE analysis along with their frequency (bracketed values) relative to the most frequent one in each case are as follows: (i) Periplasmic gate: S401 (1), S397 (0.8), S70 (0.7), L69 (0.6), and Y270 (<0.1); (ii) Central bottleneck: Y393 (1), Y266 (0.4), S73 (0.3), W138 (0.3), Y76 (0.1), H165 (0.1), Y42 (<0.1), Q134 (<0.1), Y270 (<0.1), I72 (<0.1), and Y38 (<0.1); (iii) Cytoplasmic gate: L373 (1), V146 (0.6), I157 (0.2), L370 (0.1), M145 (<0.1), and V158 (<0.1). **d** Projection of the same lines shown in **c** onto the 2D space of Image Index and Pore Radius. The error bars represent the standard deviation (see Analysis techniques in Methods).

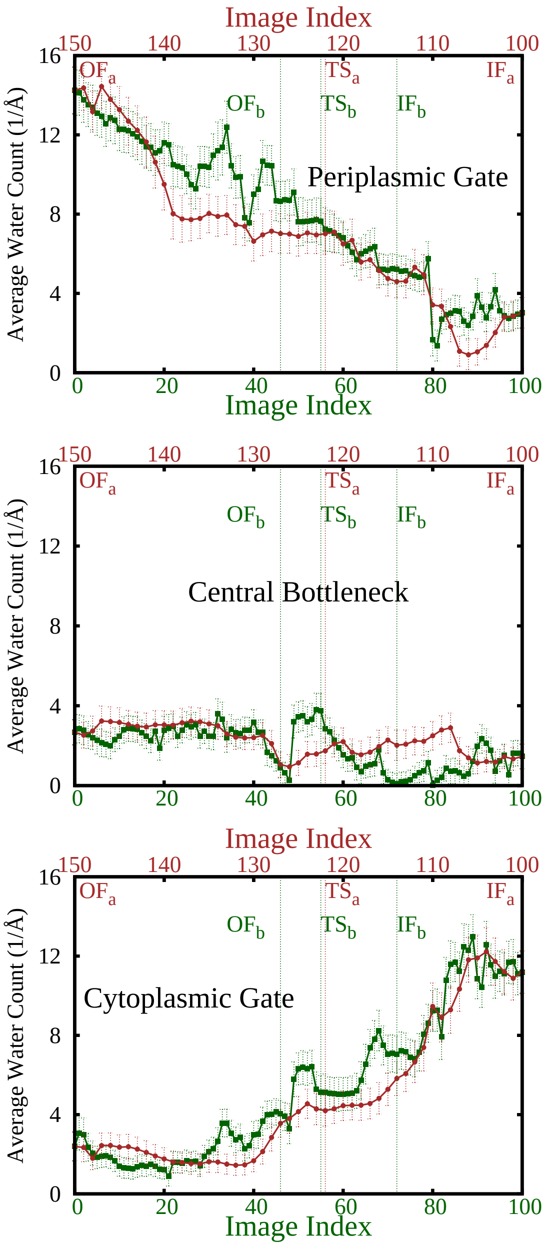

Supplementary Figure 8: **Lumen water content analysis.** Average water count at the periplasmic gate, central bottleneck, and cytoplasmic gate, estimated for different images. The definitions of the gates and the central bottleneck are similar to those used for pore radius (Fig. 3 and Supplementary Fig. 7). A bin size of 1 Å along z was used to generate the histograms used for the analysis. The error bars represent the standard deviation (see Analysis techniques in Methods).

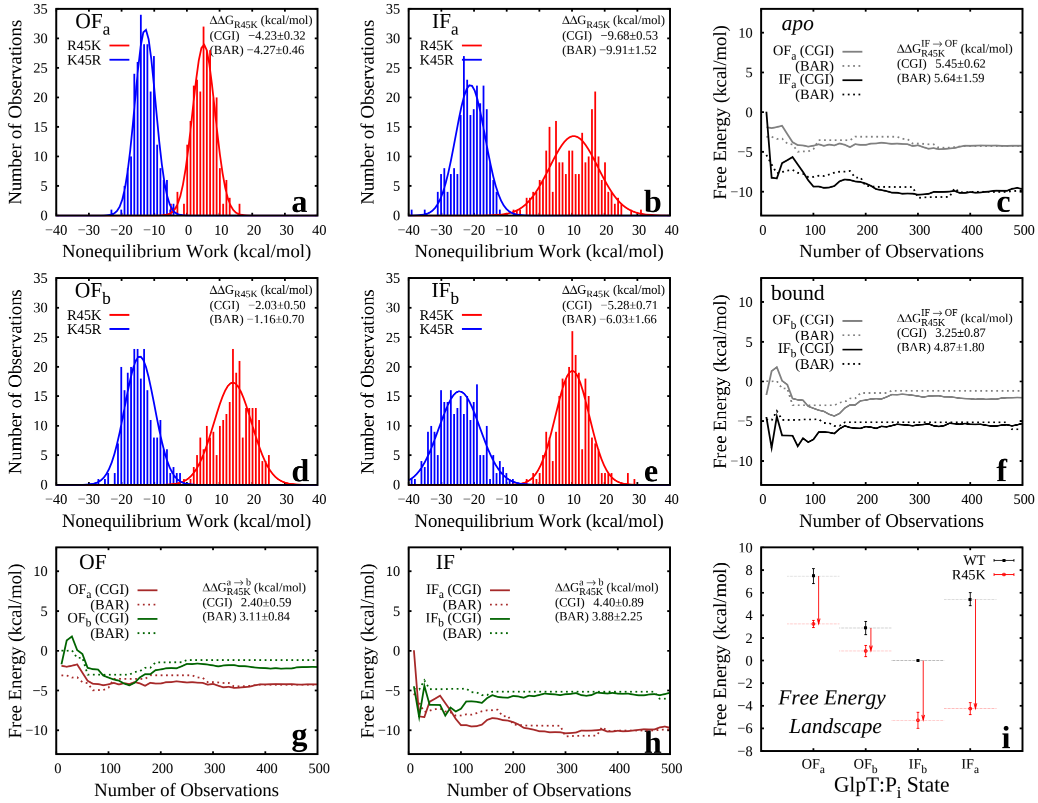

Supplementary Figure 9: **Energetic effects of R45K mutation.** **a,b,d,e** Forward (R45K; red) and reverse (K45R; blue) nonequilibrium work distributions obtained from FGTI [8, 9] simulations for four major simulated states of GlpT:Pi complex including OF$_a$, IF$_a$, OF$_b$, and IF$_b$ (see Nonequilibrium alchemical free energy calculations in Methods and Supplementary Note 2 for simulation details). **c,f,g,h** Relative conformational (**c,f**) and binding (**g,h**) free energies of R45K mutant with respect to the WT protein for different states of GlpT:Pi complex including *apo* (**c**) and bound (**f**) states for conformational free energies and OF (**g**) and IF (**h**) for binding free energies. The free energies are estimated employing both CGI (solid lines) and BAR (dotted lines) methods using different number of FGTI simulations (x axis) while the same number of forward and reverse simulations are used for each free energy estimate. **i** The free energies associated with WT OF$_a$, IF$_a$, OF$_b$, and IF$_b$ states (black dotted lines) estimated from Simulation Set 13 (Fig. 1e) used along with CGI-based relative free energy estimates (red arrows) to find the free energies associated with OF$_a$, IF$_a$, OF$_b$, and IF$_b$ states of R45K mutant (red dotted lines). Based on the relative population of IF and OF states, the effective relative binding free energy was found to be dominated by the relative binding free energy of the highly populated IF state (*i.e.*, $\Delta_{IF,OF}$ is negligible), which is 4.40±0.89 kcal/mol (**h**). Note that binding affinity of wild-type GlpT to G3P is only ∼2.5 times higher than that to P$_i$ [10] while our calculations show more than 3 orders of magnitude decrease in GlpT's binding affinity to P$_i$ due to R45K mutation. For a more direct comparison to R45K mutant experimental results [11], however, our simulations may be repeated with G3P. see Nonequilibrium alchemical free energy calculations in Methods for error estimates.

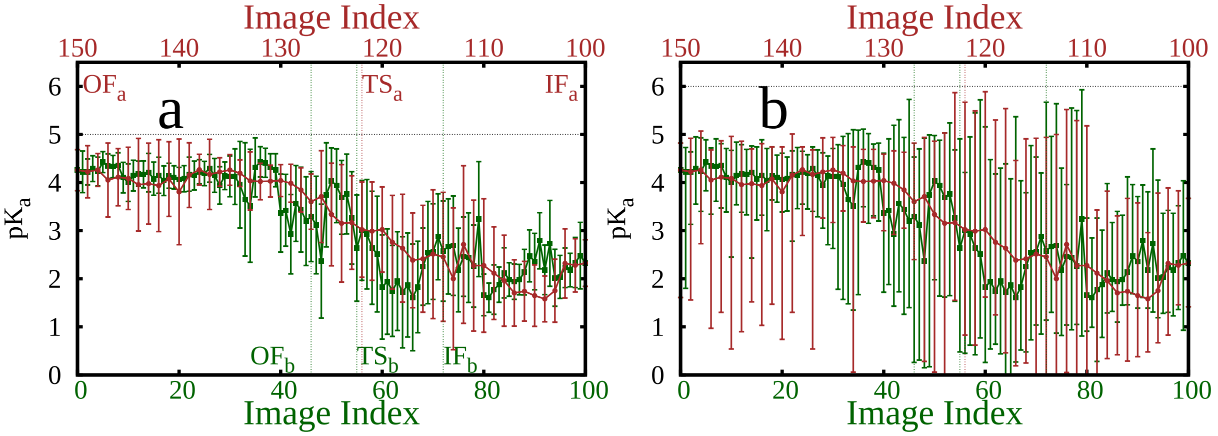

Supplementary Figure 10: **H165 pK$_a$ predictions.** Predicted pK$_a$ values of binding site residue H165 for different images averaged over 1% of all collected snapshots, (*i.e.*, 80 conformations for each image, collected every 0.5 ns) estimated using PROPKA 3.1 [12, 13]. The error bars show the sample standard deviations (**a**) and the range in which predicted pK$_a$'s were observed (**b**). MolProbity 4.1 [14] was used to determine the protonation state of H165 for a select number of conformations at IF$_a$, IF$_b$, TS$_b$, OF$_b$, OF$_a$, and TS$_a$ states which indicated a N$^\delta$-only protonation—which was also predicted for GlpT crystal structure (PDB:1pw4) and was the basis of our initial model and all simulations performed. We note that the possibility of H165 protonation or proton transfer between H165 N$^\delta$ and N$^\epsilon$ as well as P$_i$ cannot be ruled out, particularly, if other conformational pathways not sampled in our simulations are accessible (or become accessible upon proton transfer). The error bars represent the standard deviation (see Analysis techniques in Methods).

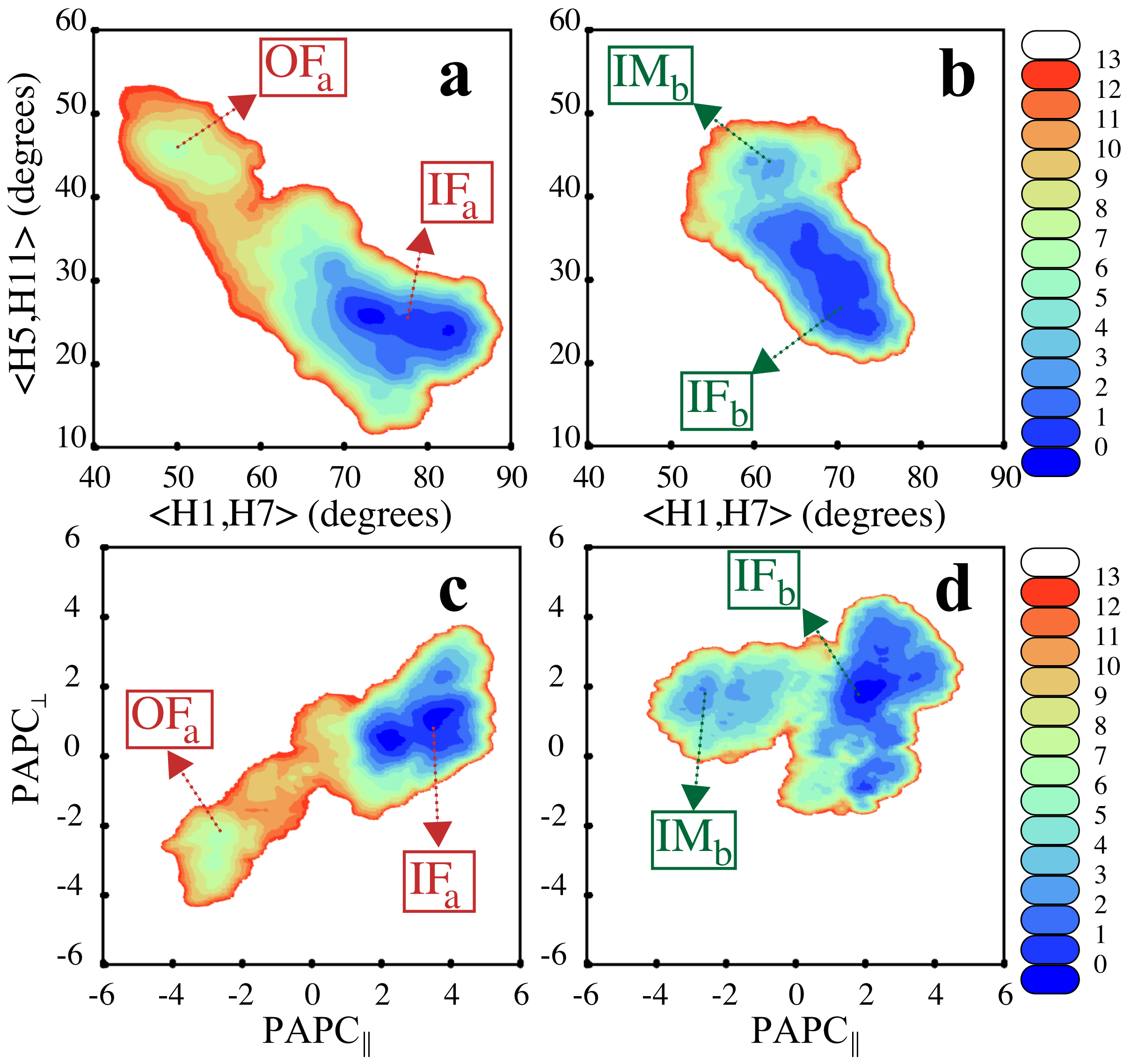

Supplementary Figure 11: **Comparison of initial *apo* and P_i-bound state transition BEUS simulations.** PMF reconstructed in representative 2D spaces from (Q_1, Q_7) based on Simulation Sets 1 and 8 sampling $IF_a \leftrightarrow OF_a$ transition (left panels) and $IF_b \leftrightarrow OF_b$ transition (right panels), respectively. **a,b** PMF in terms of different interhelical angles (see Fig. 2). In the P_i-bound simulations, the protocol which only involves protein conformational changes does not result in a stable OF_b GlpT. The conformation denoted as IM_b (an intermediate P_i-bound state) is a local minimum which was used as a reference structure for follow-up Simulation Sets 9 and 10. **c,d** PMF in terms of two collective variables constructed from principal-axis principal components (PAPC) of Simulation Sets 1 and 8. $PAPC_{\|}$ is the first PAPC of Simulation Set 8 while the first PAPC of Simulation Set 1 is decomposed to $PAPC_{\|}$ and $PAPC_{\perp}$. It reveals that the (Q_1, Q_7) based protocol fails to induce a conformational change along $PAPC_{\perp}$.

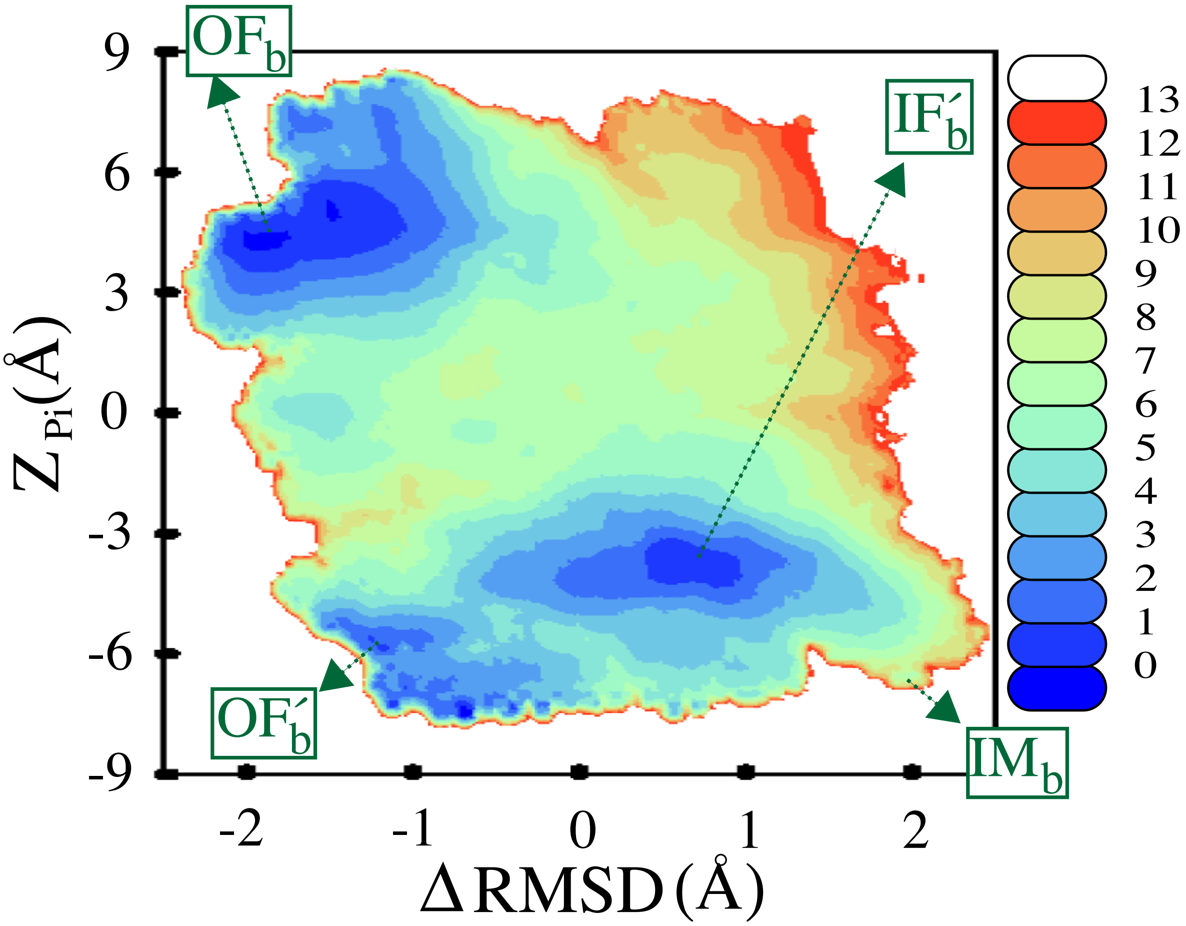

Supplementary Figure 12: **Coupling between substrate translocation and protein conformational changes.** PMF reconstructed from the 2D BEUS simulation (Simulation Set 10) sampling part of the IF$_b$ ↔OF$_b$ transition. $\Delta RMSD = RMSD_{OF_b} - RMSD_{IM_b}$ and Z_{Pi} represent the conformational change of the protein and translocation of the phosphate within the binding site, respectively. IF$'_b$ and OF$'_b$ conformations were two local minima resulted from this set of simulations. The former is a not-fully-relaxed IF$_b$ conformation and the latter is a locally stable P$_i$-bound OF structure which may represent a second pathway for the IF$_b$ ↔OF$_b$ transition.

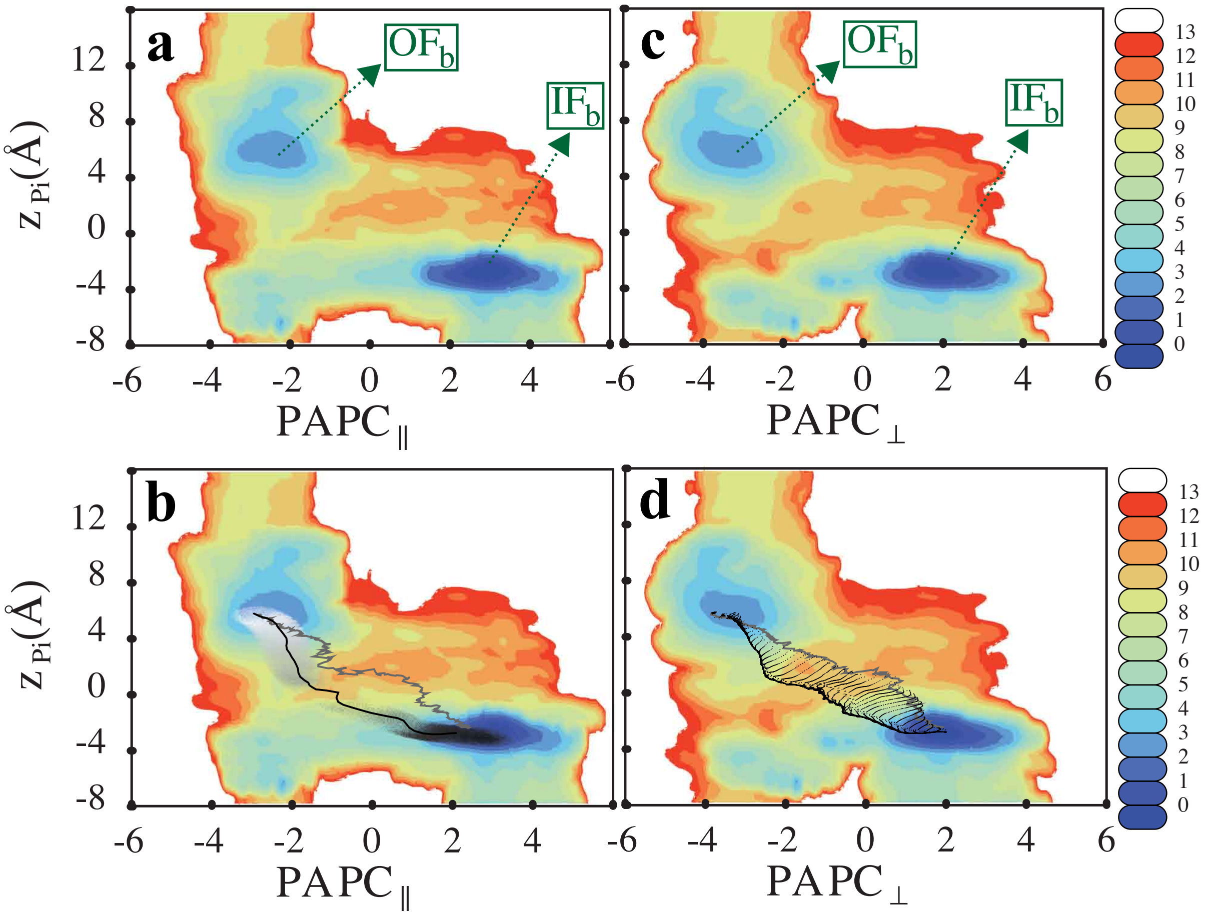

Supplementary Figure 13: **Post-hoc string method (PHSM).** PMF reconstructed from several patches of BEUS simulations including Simulation Sets 1, 4, 6, and 8–10 in terms of **a,b** (PAPC$_\parallel$,Z_{Pi}) and **c,d** (PAPC$_\perp$,Z_{Pi}). See Fig. S11 for the definition of PAPCs. **b,d** Visual illustration of the PHSM algorithm. Gray line is the projection of the initial pathway (resulted from a non-parametric version of lowest free energy path algorithm [15]; see Supplementary Note 3) onto the given 2D space. The solid black line is the projection of the converged PHSM pathway onto the same space. The gray-scale spectra in **b** show the individual samples projected onto the given space colored based on their converged Voronoi cell ranging from white (OF$_b$ minimum) to black (IF$_b$ minimum). The dotted lines in **d** show the evolution of the PHSM pathway prior to convergence.

Supplementary Table 1: List of the BEUS and SMwST simulations.

**Simulation Set**	**Transition**	**Technique**	**Initial Conformations**	**Exchange Rate** *
1	$\mathrm{IF}_a \leftrightarrow \mathrm{OF}_a$	BEUS	NE † (crystal structure ‡)	32%
2		SMwST	Set 1 (using PHSM)	-
3		BEUS	Set 2 (using PHSM)	22%
4	$\mathrm{IF}_a \leftrightarrow \mathrm{IF}_b$	BEUS	IF_a (from Set 1)	35%
5		BEUS	Set 4 (using PHSM)	23%
6	$\mathrm{OF}_a \leftrightarrow \mathrm{OF}_b$	BEUS	OF_a (from Set 1)	34%
7		BEUS	Set 6 (using PHSM)	23%
8	$\mathrm{IF}_b \leftrightarrow \mathrm{OF}_b$	BEUS	NE (Set 4)	23%
9		BEUS	IM_b § (from Set 8)	36%
10		BEUS	NE (Set 9)	16%
11		SMwST	Sets 4,6,8,9,10 (using PHSM)	-
12		BEUS	Set 11 (using PHSM)	51%
13	Full Cycle	BEUS	Sets 3,5,7,12 (using PHSM)	30%

*Only the nearest neighbor exchange rate is reported.

†NE denotes nonequilibrium pulling simulations whose initial conformations are given in the brackets. For the description of these simulations in each case see Methods and Supplementary Note 1.

‡An equilibrated model (IF_a^*) based on the crystal structure of GlpT was available from previous studies [16, 17] which was used as the initial conformation for these nonequilibrium pulling simulations (see Methods and Supplementary Note 1).

$^\S\mathrm{IM}_b$ is an "intermediate" conformation which appeared as a local minimum in Set 8.

Supplementary Table 2: Autocorrelation time τ_{ac} associated with different collective variables obtained from the final BEUS simulation (Set 13).

	$\tau_{\mathbf{ac}}$ (ps)		$\tau_{\mathbf{ac}}$ (ps)		$\tau_{\mathbf{ac}}$ (ps)		$\tau_{\mathbf{ac}}$ (ps)
Image Index	1039±64*	QPC_1	741±51	PC_1	1312±67	$\chi_1(H165)$	200±44
Z_{Pi}	770±68	QPC_2	528±40	PC_2	1242±73	$D_{min}(K46-D274)$	625±45
Q_7	668±45	QPC_3	503±40	PC_3	1457±73†	$D_{min}(R45-Pi)$	865±68
Q'_7	612±47	QPC_4	612±39	PC_4	1359±71	$D_{min}(R269-Pi)$	1076±73

*The mean and standard deviation of the mean, reported based on 150 independent trajectories/replicas.
†The largest autocorrelation time among ∼200 quantities whose autocorrelation times were calculated.

Supplementary Table 3: Pairwise C^{α}-RMSD of different states in Å.

	**OF**$_a$	**OF**$_b$	**TS**$_b$	**IF**$_b$	**IF**$_a$	**TS**$_a$
**OF**$_a$	0.00 ± 0.58*	2.26 ± 0.54	3.34 ± 0.59	3.83 ± 0.58	4.42 ± 0.57	2.06 ± 0.63
**OF**$_b$	2.26 ± 0.54	0.00 ± 0.48	1.91 ± 0.54	3.09 ± 0.53	4.17 ± 0.57	3.01 ± 0.57
**TS**$_b$	3.34 ± 0.59	1.91 ± 0.54	0.00 ± 0.60	1.52 ± 0.58	3.70 ± 0.58	2.85 ± 0.63
**IF**$_b$	3.83 ± 0.58	3.09 ± 0.53	1.52 ± 0.58	0.00 ± 0.57	2.27 ± 0.56	2.71 ± 0.62
**IF**$_a$	4.42 ± 0.57	4.17 ± 0.57	3.70 ± 0.58	2.27 ± 0.56	0.00 ± 0.55	2.65 ± 0.61
**TS**$_a$	2.06 ± 0.63	3.01 ± 0.57	2.85 ± 0.63	2.71 ± 0.62	2.65 ± 0.61	0.00 ± 0.66

*The mean value of C^{α}-RMSD between the two images representing the given states along with the standard deviation estimated using principal component analysis (see Supplementary Fig. 6).

Supplementary Note 1

Sampling Protocol Details

IF↔OF transition of *apo* GlpT. The crystal structure of GlpT is in the IF state, and no OF conformation is currently available experimentally. We have recently proposed a knowledge-based empirical approach for inducing large-scale conformational changes which can be used for generating unknown states [18]. This approach differs from conventional methods (e.g., targeted MD [19] or homology modeling) in that it allows for a more systematic exploration of the configuration space using many different biasing protocols. The generated pathways are compared based on (i) the amount of nonequilibrium work required to induce the desired conformational change, and (ii) a set of knowledge-based criteria on the quality of the pathway. The relevance of the nonequilibrium work is due to the fact that the optimum pathway will be later used for free energy calculations and high work values indicate sampling irrelevant pathways (including the end state) and/or slow convergence [18, 20]. In practice, we have observed that suboptimal biasing protocols could result in undesired distortion of the protein; however, such protocols often require high work values as well.

Alternating access mechanism is one such criterion in that no intermediate should be generated which is accessible to both sides of the membrane simultaneously. Stability of the generated state (OF state in the case of GlpT) is another criterion (i.e., the generated state should remain open to the periplasm and closed to the cytoplasm after removing the bias). Some of the protocols used to induce the IF→OF transition include: (i) homology-model based targeted MD [19] using the structure of FucP transporter, a GlpT homolog crystallized in the OF state [4], (ii) orientation based pulling of TM helices towards a repeat-swapped model of OF GlpT constructed based on the inverted-topology repeat hypothesis [21, 22, 23], (iii) imposing orientation changes on NTD and CTD bundles such that an orientation quaternion was defined

for each bundle, and a 10° rotation around the axis parallel to both membrane and the pseudosymmetry plane was imposed on both domains in the opposite direction (from the periplasmic side), and (iv) imposing orientation changes on specific helices of NTD and CTD bundles including (a) H1/H3/H4/H5 and H7/H9/H10/H11, (b) H1/H3 and H7/H10 and (c) H1 and H7 only. The latter was the most successful protocol based on the criteria briefly described above. The optimum protocol was thus designed by using (Q_1, Q_7) collective variables and fine-tuning the force constant to result in the lowest total work [18]. The protocol was repeated several times to examine its reproducibility.

The alternating access mechanism was examined by monitoring the water content and the pore size of the lumen (similar to the analysis shown in Fig. 3 and Supplementary Figs. 7,8). The RMSD with respect to FucP crystal structure [4], which is in the OF state, was monitored during the IF→OF simulation as well which shows a nearly linear decrease from ∼6.5 (for IF_a^*) to ∼3.2 Å during the nonequilibrium pulling simulations (Supplementary Fig. 1a). A similar trend is observed for the RMSD with respect to the XylE crystal structure [5], which is also in the OF state, while the trend is reversed when the RMSD is calculated with respect to the GlpT crystal structure [3], which is in the IF state (Fig. S1A). Comparing these global RMSD changes to those measured for the two NTD and CTD subdomains reveal that the two subdomains stay more or less intact while the protein undergoes a large-scale global conformational change (Supplementary Fig. 1b). This observation, which is in line with the rocker-switch mechanism [3], also rules out the possibility of any major distortion in the protein during the transition. The stability of the resulting OF state was examined by performing a 50-ns unbiased simulation (Supplementary Figs. 1a,b). The equilibrium simulation of OF state was extended for another 250 ns which confirmed the relative stability of the model (Supplementary Fig. 1c).

We performed a set of BEUS simulations based on the optimum protocol and transition pathway (Simulation Set 1). 12 windows/replicas were used with centers defined in the (Q_1, Q_7)

space. The initial conformations were selected from the nonequilibrium and subsequent equilibrium simulations discussed above and shown in Figs. S1A,B. The force constant and the window centers were empirically adjusted to result in a roughly similar rate of exchange between neighboring windows (ranging between 26% to 40% in production runs). The exchange was only attempted between the immediate neighboring windows (using a deterministic odd-even scheme [24]) with an interval of 2 ps for each unique pair. The window centers (in terms of angular deviation from the reference structure IF_a^*) for window $i = 0, \ldots, 11$ were 1, 2, 3, 4, 4.8, 5.5, 6, 6.5, 7.5, 8.1, 9, and 10°, with force constants 400, 400, 500, 450, 500, 550, 600, 500, 500, 500, 500, and 600 kcal/(mol×rad^2), respectively. The free energy map confirmed the relevance of the transition pathway by showing a local low-energy minimum for the OF state and a relatively low barrier for the transition (Supplementary Fig. 11a). The last conformations from the two windows associated with IF_a and OF_a minima were equilibrated for 50 ns with no bias and used as initial conformations for the binding/unbinding BEUS simulations discussed below. The OF_a state was equilibrated for an additional 100 ns to examine its stability (Supplementary Fig. 1d).

Substrate binding/unbinding in the IF and OF states. The first set of substrate binding simulations (Simulation Set 4) was performed by placing the phosphate at different Z_{Pi} positions along the lumen within the equilibrated IF_a, replacing the closest water molecule. Two Cl^- ions were removed from the bulk to keep the system neutral and the system was minimized for 2,000 steps. The window center (force constant) for window $i = 0, \ldots, 29$ is -45 (0.002), -42 (0.002), -35 (0.002), -30 (0.05), -26.5 (0.05), -25 (0.05), -24 (0.05), -23 (1), -22 (0.5), -21 (0.5), -20 (0.5), -19 (0.5), -18 (1), -17.5 (2), -17 (3), -16.5 (4), -16 (3), -15.5 (2), -15 (1), -14 (1), -13 (1), -12 (1), -11 (1), -10.5 (1), -10 (1), -9.5 (1), -9 (1), -8.5 (1.5), -8.3 (3), and -8.15 Å (6 kcal/(mol×Å^2)), respectively. Note that the reported window centers are $Z_{Pi} - \overline{\Delta Z}$. The

exchange rate between the neighboring windows ranged between 14 to 50%. The exchange was attempted between each window and its first, second, or third neighboring window from each side using a deterministic scheme with an interval of 6 ps for each unique pair. A similar set of BEUS simulations was performed for the OF conformation (Simulation Set 6). The window center (force constant) for window $i = 0, \ldots, 29$ is 35 (0.01), 31.5 (0.01), 28 (0.01), 25 (0.01), 23 (0.01), 20 (0.015), 17 (0.025), 15 (0.045), 13 (0.055), 11 (0.075), 10 (0.095), 8.5 (0.1), 7 (0.12), 6 (0.14), 4 (0.12), 2.5 (0.1), 1.5 (0.1), 0 (0.02), -1.5 (0.1), -2 (0.15), -2.5 (0.07), -3.5 (0.1), -4.5 (0.12), -5 (0.1), -5.5 (0.09), -6 (0.07), -6.5 (0.1), -7.5 (0.07), -8.15 (0.1), and -9 Å (0.1 kcal/(mol$\times$Å^2)). The exchange rate between the neighboring windows ranged between 17 to 41%. The final conformations of the windows associated with the minima IF_b and OF_b from these simulations were equilibrated for 50 ns with no bias. The OF_b conformation was equilibrated for an additional 100 ns to examine its stability (Fig. 1e).

IF$\leftrightarrow$OF transition in the P_i-bound state. We first repeated the protocol based on the (Q_1,Q_7) collective variables used for the IF_a $\leftrightarrow$$OF_a$ transition. The nonequilibrium simulations required a lower work; however, the resulting conformation did not stay open (to the periplasmic side of the membrane) in following unbiased simulations. We also repeated the BEUS simulations based on this protocol (Simulation Set 8) which showed the resulting conformation (IM_b) was perhaps a transient state (Supplementary Fig. 11). We used 24 replicas to cover the same space in (Q_1, Q_7) covered in our *apo* BEUS simulations (Simulation Set 1). An empirical approach (similar to that used in Simulation Set 1) was employed to choose the centers and force constants which resulted in an exchange rate of 12–35% between neighboring replicas. We observed a significant difference in the binding site conformation of the IF_b and OF_b states generated from Simulation Sets 4 and 6, respectively. The IM_b conformation obtained from Simulation Set 8 (Supplementary Fig. 11) was used to initiate a new set of BEUS simulations to capture the

movement of the substrate within the binding site (Simulation Set 9). Using Z_{Pi} as the reaction coordinate (similar to Simulation Sets 4 and 6 discussed above), we sampled 15 windows with centers ($Z_{Pi} - \overline{\Delta Z}$) at 5.5, 3.0, 1.7, 0.5, -1.0, -2.2, -2.6, -3.2, -3.6, -4.2, -4.7, -5.1, -5.8, -6.7, and -8.7 Å along Z_{Pi} with force constant ranging between 0.1 to 0.2 kcal/(mol×Å^2) with an exchange rate of 23–47$\%$. There was no significant conformational change observed in these simulations and could not generate a stable OF$_b$ conformation. The conformations resulted from Simulation Set 9 were driven towards the OF$_b$ conformation using a 5-ns nonequilibrium simulation of force constant 100 kcal/(mol×Å^2) based on collective variable $\Delta RMSD$ (repeated for all 15 replicas). We then used the generated conformations in a 2D BEUS simulation to capture the coupling between the substrate translocation within the binding site and the global conformational change of the protein (Simulation Set 10). Supplementary Figure 12 shows the PMF generated from this set of simulations in reaction coordinate space $(\Delta RMSD, Z_{Pi})$.

Refining the BEUS protocols. The complications encountered in our attempts to design a BEUS protocol for reconstructing the IF$_b$ $\leftrightarrow$OF$_b$ transition using a single set of reaction coordinates led us develop a novel algorithm to "patch" together a number of BEUS simulations in order to design an improved BEUS protocol. This approach involves PHSM and SMwST techniques. We thus combined all reweighted samples (see Methods for reweighting scheme) from different BEUS patches including Simulation Sets 4, 6, 8, 9, and 10 to build a sample set covering a "continuous" space which, among many conformations, includes both IF$_b$ and OF$_b$ states. Supplementary Figure 13 shows the PMF in a select number of 2D spaces generated based on this reweighted sample set. Upon the completion of the patching procedure, the principal curve was constructed using the PHSM algorithm whose projections onto two select spaces is shown in Supplementary Fig. 13 (see Methods). The metric used for this algorithm included the following collective variables: (i) Z_{Pi}; (ii) first 8 C$^\alpha$-based principal components; (iii) first 8

principal-axis principal components (PAPC; see Supplementary Fig. 5); (iv) inter-residue D_{min} of following salt-bridge forming pairs K46-E299, K46-D274, R269-E299, R45-E299, and R45-D274; (v) D_{min} of P_i and the following residues: R269, K80, K46, R45, H165, Y38, Y42, Y76, Y266, Y270, and Y393; (vi) side-chain dihedral angle χ_1 of all 13 residues named in (iv) and (v). A distance was defined in the 46-dimensional collective variable space of (i) to (vi) using a diagonal metric matrix with values $1/\text{Å}^2$, $10^2/\text{Å}^2$, 10^4, $10^2/\text{Å}^2$, $1/\text{Å}^2$, and $0.04/(1°)^2$ for elements in (i) to (vi), respectively (considering minimum image criterion for collective variables with periodic boundary condition). The weights (metric matrix elements) were chosen roughly based on the relative variance of collective variables (see Supplementary Note 3 for alternative definitions). A 1D string was defined based on 50 image centers, initially generated using a non-parametric version of the Lowest Free Energy Pathway (LFEP), originally introduced in its parametric version in Ref. [15]. The same metric defined above was used for the non-parametric LFEP (NPLFEP) algorithm. (NP)LFEP method does not require an initial pathway; however, the dependence of the (NP)LFEP algorithm to its parameters is not smooth and drastically different pathways could be resulted by small changes in the parameters. Several initial pathways were generated using the the NPLFEP algorithm to seed the PHSM algorithm. Interestingly, the PHSM algorithm more or less converges to the same pathway regardless of its initial pathway as long as the end points, the tube thickness, and the number of images are kept constant. The tube thickness $\epsilon = 7$ was used in the PHSM algorithm which gives the smoothest pathway when compared to other results based on $\epsilon = 1, \ldots, 10$. The same parameters were used to extract the PHSM pathways from Simulation Set 1 (for $IF_a \leftrightarrow OF_a$ transition), Simulation Set 4 (for $IF_a \leftrightarrow IF_b$ transition), and Simulation Set 6 (for $OF_a \leftrightarrow OF_b$ transition); however, the number of images used was 30 for the two latter cases. In the former case, the collective variables based on P_i were excluded from the metric since the substrate was not present in the simulations. Two of the PHSM pathways generated above for $IF_a \leftrightarrow OF_a$ and $IF_b \leftrightarrow OF_b$ transitions were further

refined using the SMwST method (Simulation Sets 2 and 11). 100 iterations of runtime 10 ps were performed with restraining and releasing stages of runtime 5 ps. The collective variables used were $\{Q\}$ and $(\{Q\}, Z_{Pi})$ for the *apo* and bound simulations. A Euclidean metric was used; however, Z_{Pi} was scaled by a $0.1/\text{Å}$ factor if the metric involved Z_{Pi}. SMwST simulations were initiated using 50 conformations which represented the closest samples to the PHSM image centers. 20 copies of each image was used in the simulations which included 1000 replicas, in total. The force constants 100 kcal/(mol×Å^2) and 10000 kcal/(mol×rad^2) were used for the harmonic potentials based on Z_{Pi} and $\{Q\}$, respectively. In both simulations, no significant changes were observed in the image centers after $\sim$20 iterations. The final centers and a representative conformation from each image were used to initiate the follow-up BEUS simulations. For the binding/unbinding simulations we used PHSM results to initiate the following BEUS simulations since the conformational changes were not as significant/slow as the IF↔OF transition. The follow-up BEUS simulations including Simulation Sets 3, 5, 7, and 12 used $(\{Q\}, Z_{Pi})$ as the collective variables except in the former case which did not include the Z_{Pi} collective variable due to the absence of the substrate in the simulations.

PHSM algorithm was used with the same parameters mentioned above to extract a cyclic pathway (*i.e.*, a closed curve in $(\{Q\}, Z_{Pi})$ space) of 140 images representing the entire thermodynamic cycle. Collective variable Z_{Pi} was ignored in PHSM algorithm when the distance was calculated between conformations lacking P_i (*i.e.*, *apo* simulations). The converged PHSM pathway was used to initiate the final set of BEUS simulations, *i.e.*, Simulation Set 13. A phosphate was added at a given Z_{Pi} (see below) to the conformations lacking the substrate using the same protocol described above (replacing the closest water, removing two Cl^- ions, and minimization). For the images representing the IF$_a$ ↔OF$_a$ leg of the cycle, the phosphate was added at $Z_{Pi} \approx -35\,Å$ (images 102 to 147), which is far enough from the protein not to interact with it. Taking advantage of the periodic boundary condition along z axis and in order to place

the images along a truly closed curve in the $(\{Q\}, Z_{Pi})$ space, we generated 10 copies of the conformation representing OF_a state and placed the phosphate at Z_{Pi} covering the range from -35 Å to the lower boundary and from the upper boundary to 40 Å (images 148 to 150 and 1 to 7). These additional images along with 140 images extracted from the PHSM algorithm (*i.e.*, 150 images in total), were used as initial conformations for the final simulation set. The force constants 2 kcal/(mol$\times$Å^2) and 2,000 kcal/(mol$\times$rad^2) were used for the restraining harmonic potentials imposed on Z_{Pi} and $\{Q\}$, respectively. A periodic exchange was attempted between replicas i and j, for which $\min(||i-j-150|, |i-j|, |i-j+150|)$ is 1 or 2 (*i.e.*, first and second nearest neighbors with periodic boundary condition), with an interval of 20 ps for each unique pair.

Supplementary Note 2

Nonequilibrium alchemical free energy calculations.

In Methods we described how the relative free energy of OF_a, IF_a, OF_b, and IF_b states due to R45K mutation were estimated. Denoting the free energy change of state X due to R45K mutation as $\Delta\Delta G^{X}_{WT\rightarrow R45K}$ (or $\Delta\Delta G^{X}_{R45K}$, for brevity), in which X is OF_a, IF_a, OF_b, or IF_b, the relative binding ($a \rightarrow b$) free energy of the R45K mutant with respect to the wild-type (WT) protein, $\Delta\Delta G^{a\rightarrow b}_{R45K}$ would be $\Delta\Delta G^{IF_a\rightarrow IF_b}_{R45K} = \Delta\Delta G^{IF_b}_{R45K} - \Delta\Delta G^{IF_a}_{R45K}$ and $\Delta\Delta G^{OF_a\rightarrow OF_b}_{R45K} = \Delta\Delta G^{OF_b}_{R45K} - \Delta\Delta G^{OF_a}_{R45K}$ for IF and OF states, respectively. Similarly, the relative conformational ($IF \rightarrow OF$) free energy of the R45K mutant with respect to the WT protein, $\Delta\Delta G^{IF\rightarrow OF}_{R45K}$ would be $\Delta\Delta G^{IF_a\rightarrow OF_a}_{R45K} = \Delta\Delta G^{OF_a}_{R45K} - \Delta\Delta G^{IF_a}_{R45K}$ and $\Delta\Delta G^{IF_b\rightarrow OF_b}_{R45K} = \Delta\Delta G^{OF_b}_{R45K} - \Delta\Delta G^{IF_b}_{R45K}$ for *apo* and bound states, respectively. The free energies associated with WT OF_a, IF_a, OF_b, and IF_b states estimated from Simulation Set 13 (Fig. 1e) can be used to find the free energies associated with OF_a, IF_a, OF_b, and IF_b states of R45K mutant. However, as noted in the main manuscript the free energy difference between the IF_a and IF_b (or OF_a and OF_b) states based on the PMF alone does not give the standard binding free energies of IF (or OF) state. Having the relative population of WT IF and OF states from the PMF (Fig. 1e) and the FGTI-based relative free energies, one may estimate the relative binding free energy due to the R45K mutation in an effective manner as follows:

$$\Delta\Delta G^{a\rightarrow b}_{R45K} = -\beta^{-1}\log\frac{P_b(IF)\exp(-\beta\Delta\Delta G^{IF_b}_{R45K}) + P_b(OF)\exp(-\beta\Delta\Delta G^{OF_b}_{R45K})}{P_a(IF)\exp(-\beta\Delta\Delta G^{IF_a}_{R45K}) + P_a(OF)\exp(-\beta\Delta\Delta G^{OF_a}_{R45K})}, \quad \text{(S1)}$$

in which $P_a(IF) \doteq \frac{P_{WT}(IF_a)}{P_{WT}(IF_a)+P_{WT}(OF_a)}, \ldots$, and $P_{WT}(X) \propto \exp(-\beta\Delta\Delta G_X)$ is estimated from the free energy profile in Fig. 1e. One can show $\Delta\Delta G^{a\rightarrow b}_{R45K} = \Delta\Delta G^{IF_a\rightarrow IF_b}_{R45K} + \Delta_{IF,OF}$, in which $\Delta_{IF,OF}$ (defined below) turned out to be negligible in our calculations,

$$\Delta_{IF,OF} = -\beta^{-1}\left(\log\frac{1+\exp(-\beta(\Delta\Delta G^{IF_b\rightarrow OF_b}_{R45K}+\Delta\Delta G_{IF_b\rightarrow OF_b}))}{1+\exp(-\beta(\Delta\Delta G^{IF_a\rightarrow OF_a}_{R45K}+\Delta\Delta G_{IF_a\rightarrow OF_a}))} + \log\frac{1+\exp(-\beta\Delta\Delta G_{IF_a\rightarrow OF_a})}{1+\exp(-\beta\Delta\Delta G_{IF_b\rightarrow OF_b})}\right). \quad \text{(S2)}$$

Supplementary Note 3

Post-hoc string method algorithm

Here we present a more detailed description of PHSM algorithm, briefly described in Methods. The description is kept general here while the particular parameters used in our analysis are already discussed above in Sampling Protocol Details. The PHSM algorithm requires a relatively large set of samples $\{\mathbf{x}^t\}$, along with their weights $\{\omega^t\}$ determined using a non-parametric reweighting scheme (*e.g.*, Eq. (8) or (11) in Methods). The two end conformations must be specified as well; although the algorithm will attempt to find the minima close to these conformations. In addition, there are several parameters involved which in principle should be empirically examined to determine the dependence of the final solution on them. The parameters must be chosen such that the final solution is at least locally stable. If several alternate, locally stable pathways are generated (with similar end points), one may estimate the free energy barrier associated with each path, $-k_B T \min_i \log(< \omega^t >_i)$ ($\langle . \rangle$ is defined below), and pick the one with the lowest barrier. The PHSM parameters include: (i) a metric/distance/norm $||.||$ to measure the closeness of conformations; (ii) an initial pathway of N conformations; (iii) transition tube thickness parameter ϵ; and (iv) convergence criteria including a threshold δ and a persistence number P: The particular algorithms used below for finding an initial path and reparametrization have their own parameters (*i.e.*, $\Delta_{min/max}$ and δ', respectively).

1. Metric definition:

   (a) For each conformation $\mathbf{x}^t$, measure the relevant collective variables $\boldsymbol{\zeta}^t$. For instance, $\boldsymbol{\zeta}$ could consist of all C^α atoms of protein or a set of system-specific collective variables $(\boldsymbol{\alpha}, \boldsymbol{\beta}, \ldots)$ in which $\boldsymbol{\alpha}$, $\boldsymbol{\beta}$, ... could be one- or multidimensional collective variables such as distance, displacement vector, or orientation quaternion (see Supplementary Note 1 for an example).

(b) Define a metric to measure the distance of two given conformations $\boldsymbol{\zeta}$ and $\boldsymbol{\zeta}'$, $||\boldsymbol{\zeta} - \boldsymbol{\zeta}'||$. For instance, if $\boldsymbol{\zeta}$ consists of all C^{α} atoms of protein, one may define the distance between two conformations as their RMSD. Alternatively, if $\boldsymbol{\zeta}$ is $(\boldsymbol{\alpha}, \boldsymbol{\beta}, \ldots)$, one may define $||\boldsymbol{\zeta} - \boldsymbol{\zeta}'|| = w_{\alpha}||\boldsymbol{\alpha} - \boldsymbol{\alpha}'|| + w_{\beta}||\boldsymbol{\beta} - \boldsymbol{\beta}'|| + \ldots$ in which each collective variable such as $\boldsymbol{\alpha}$ has a well-defined (*e.g.*, Euclidean) metric/distance and w_{α} determines its weight or contribution to the total distance. The weights could be, for instance, proportional to $1/\langle||\boldsymbol{\alpha} - \langle\boldsymbol{\alpha}\rangle||\rangle, \ldots$ in which $\langle . \rangle$ is an average over $\{\boldsymbol{\zeta}^t\}$. An alternative definition is $||\boldsymbol{\zeta} - \boldsymbol{\zeta}'|| = (w_{\alpha}||\boldsymbol{\alpha} - \boldsymbol{\alpha}'||^2 + w_{\beta}||\boldsymbol{\beta} - \boldsymbol{\beta}'||^2 + \ldots)^{1/2}$ in which w's are proportional to $1/\langle||\boldsymbol{\alpha} - \langle\boldsymbol{\alpha}\rangle||^2\rangle, \ldots$. The latter definition was used in our PHSM analysis in which weights are roughly proportional to the inverse of the variance of collective variables (see Supplementary Note 1).

2. Choose ϵ, δ, and P. Note that the choice of ϵ and δ are dependent on the definition of metric.

3. Iteration 0: choose an initial string $\{\boldsymbol{\zeta}_i\}$ in which $i = 0, \ldots, N-1$. The following algorithm loosely resembles the lowest free energy path (LFEP) method [15], which is termed here as non-parametric lowest free energy path (NPLFEP):

   (a) Choose a representative conformation for the start (end) point, $\boldsymbol{\zeta}_0$ ($\boldsymbol{\zeta}_{end}$).

   (b) Choose a minimum and a maximum step size: Δ_{min} and Δ_{max}.

   (c) (Optional) Replace $\boldsymbol{\zeta}_0$ with $\boldsymbol{\zeta}^t$ whose weight ω^t is the highest among all $\{\boldsymbol{\zeta}^t\}$ satisfying $||\boldsymbol{\zeta}^t - \boldsymbol{\zeta}_0|| < \Delta_{max}$.

   (d) Starting with $i = 1$ and incrementing by 1 at every step, pick $\boldsymbol{\zeta}_i$ from $\{\boldsymbol{\zeta}^t\}$ such that $\Delta_{min} < ||\boldsymbol{\zeta}_i - \boldsymbol{\zeta}_{i-1}|| < \Delta_{max}$ and $||\boldsymbol{\zeta}_i - \boldsymbol{\zeta}_{end}|| < ||\boldsymbol{\zeta}_{i-1} - \boldsymbol{\zeta}_{end}||$ ($\boldsymbol{\zeta}_i$ is not unique).

   (e) (Optional) An alternative algorithm to (d) picks $\boldsymbol{\zeta}^t$ for $\boldsymbol{\zeta}_i$ in which ω^t is the highest

weight among all conformations satisfying criteria in (d).

(f) Stop (d)/(e) when $i = M$ such that $||\boldsymbol{\zeta}_M - \boldsymbol{\zeta}_{end}|| < \Delta_{max}$.

(g) If $N < M$, discard $M - N$ conformations from $\{\boldsymbol{\zeta}_i\}$ (*e.g.*, one every $|\frac{N}{N-M}|$ steps). If $N > M$, reduce Δ_{min} and/or Δ_{max} and repeat (c)-(g).

4. Iterate over the following steps until convergence criteria are satisfied:

(a) Voronoi tessellation:

i. For each $\boldsymbol{\zeta}^t$ and $\boldsymbol{\zeta}_i$, measure $D_i^t = ||\boldsymbol{\zeta}^t - \boldsymbol{\zeta}_i||$ and discard D_i^t, if $D_i^t > \epsilon$.

ii. For each t, find the smallest undiscarded D_i^t, and discard the rest.

iii. For each i, make a set of all t ($\{t\}_i$) with undiscarded D_i^t values. If the set is empty, the algorithm is failed. Some parameters need to be modified.

(b) For each image i, find its expected value $\bar{\boldsymbol{\zeta}}_i = \langle \omega^t \boldsymbol{\zeta}^t \rangle_i / \langle \omega^t \rangle_i$ in which $\langle . \rangle_i$ averages over all t in $\{t\}_i$.

(c) Reparametrization ($B\acute{e}zier$ technique): Evaluate $\boldsymbol{\zeta}'(s) = \sum_i \binom{N-1}{i} s^i (1-s)^{N-i-1} \bar{\boldsymbol{\zeta}}_i$ at N equidistant points between $s = 0$ and $s = 1$.

i. de Casteljau algorithm [25]: $\boldsymbol{\zeta}'(s)$ can be evaluated at a given s by recursively solving $\boldsymbol{\zeta}_i^{(j)}(s) = (1-s)\boldsymbol{\zeta}_i^{(j-1)}(s) + s\boldsymbol{\zeta}_{i+1}^{(j-1)}(s)$ for $j = 1, \ldots, N-1$ and $i = 0, \ldots, N-j-1$, in which $\boldsymbol{\zeta}_i^{(0)}(s) = \bar{\boldsymbol{\zeta}}_i$ and $\boldsymbol{\zeta}'(s) = \boldsymbol{\zeta}_0^{(N-1)}(s)$.

ii. To pick N equidistant points, one may start with $s_i = \frac{i}{N-1}$ to evaluate $\boldsymbol{\zeta}'(s_i)$ for all i; then move each s_i (except for $s_0 = 0$ and $s_{N-1} = 1$) by $r_i \frac{s_{i+1}-s_i}{2}$ (or $r_i \frac{s_i - s_{i-1}}{2}$) if $r_i = \frac{||\boldsymbol{\zeta}'(s_i) - \boldsymbol{\zeta}'(s_{i+1})|| - ||\boldsymbol{\zeta}'(s_i) - \boldsymbol{\zeta}'(s_{i-1})||}{||\boldsymbol{\zeta}'(s_i) - \boldsymbol{\zeta}(s_{i+1})|| - ||\boldsymbol{\zeta}'(s_i) + \boldsymbol{\zeta}(s_{i-1})||}$ is positive (or negative). Iterate until $r_i < \delta'$ for all i, in which δ' is a convergence threshold.

(d) Convergence check: Determine the change in the string with respect to previous step: $\Delta = \sum_{i=0}^{N-1} ||\boldsymbol{\zeta}_i - \boldsymbol{\zeta}_i'||$ (now all centers $\{\boldsymbol{\zeta}_i\}$ can be replaced by $\{\boldsymbol{\zeta}_i'\}$). If

$\Delta < \delta$ for P consecutive iterations, string is converged.

If the PHSM algorithm fails (step 4(a)iii), it often implies that the string has moved to an undersampled region. It is advisable to allow for a few failures (*e.g.*, F) before stopping the algorithm. This can be easily achieved since the reparametrization algorithm can generate N new centers from $N-F$ points. If the algorithm still fails, one may either increase ϵ, change the metric (*e.g.*, by reducing the dimensionality of collective variable space), or change the parameters involved in initial path generation. The number of degrees of freedom in the collective variable space is quite important. The failure of the algorithm is often due to finite sample size whose effect is larger in higher-dimensional spaces. Using system-specific collective variables and/or using dimensionality reduction techniques can provide a tool in defining lower-dimensional spaces. In the case of GlpT in which we had $\sim 10^6$ samples, we had to keep the collective variable space $\sim$50-dimensional in order to have a stable PHSM solution.

Supplementary Note 4

Data analysis

In Methods, we described how we generated the data for statistical analysis of Simulation Set 13 which involved discarding the first 10 ns of each trajectory and blocking the rest of the data into blocks of simulation time 10 ns. The block size as well as the size of the discarded data were chosen based on the estimated autocorrelation times of the time series. Our autocorrelation analysis, as shown in Supplementary Table 2, indicates that the largest autocorrelation time, τ (averaged over all replicas) is $\sim$1.5 ns. For some of the individual replicas, however, τ goes up to $\sim$3 ns. We discarded the first 10 ns which is several times larger than the largest autocorrelation time measured. We used an analytic approach to roughly estimate the size of the discarded data. Given the total sample size, $T = 50$ ns and the autocorrelation time $\tau \approx 3$ ns, the optimum discarded size, Δ to balance the error due to statistical uncertainty $(O(\sqrt{\frac{\tau}{T-\Delta}}))$ and the relaxation bias $(O(\exp(-\frac{\Delta}{\tau})))$ is determined by minimizing the total error which is the solution to the transcendental equation $(\frac{N-\Delta}{\tau} = 2^{-\frac{2}{3}} \exp(\frac{2}{3}\frac{\Delta}{\tau}))$ whose solution for Δ is approximately 10 ns. In the context of block bootstrapping, the optimum block size B can be estimated using similar approach, resulting in an analytic estimate [**?**], $B \approx \tau(2T/\tau)^{1/3}$, which is also approximately 10 ns.

Supplementary References

[1] Dahl, A. C. E. & Sansom, M. S. P. Bendix: intuitive helix geometry analysis and abstraction. *Bioinformatics* **28**, 2193–2194 (2012).

[2] Sugeta, H. & Myiazava, T. General method for calculating helical parameters of polymer chains from bond length, bond angles, and internal rotation angles. *Biopolymers* **5**, 673–679 (1967).

[3] Huang, Y., Lemieux, M. J., Song, J., Auer, M. & Wang, D.-N. Structure and mechanism of the glycerol-3-phosphate transporter from *Escherichia coli*. *Science* **301**, 616–620 (2003).

[4] Dang, S. *et al.* Structure of a fucose transporter in an outward-open conformation. *Nature* **467**, 734–738 (2010).

[5] Sun, L. *et al.* Crystal structure of a bacterial homologue of glucose transporters GLUT1–4. *Nature* **490**, 361–366 (2012).

[6] Roberts, E., Eargle, J., Wright, D. & Luthey-Schulten, Z. MultiSeq: Unifying sequence and structure data for evolutionary analysis. *BMC Bioinformatics* **7**, 382 (2006).

[7] Smart, O. S., Neduvelil, J. G., Wang, X., Wallace, B. A. & Sansom, M. S. P. HOLE: A program for the analysis of the pore dimensions of ion channel structural models. *J. Mol. Graphics* **14**, 354–360 (1996).

[8] Seeliger, D., Buelens, F. P., Goette, M., de Groot, B. L. & Grubmüller, H. Towards computional specificity screening of DNA-binding proteins. *Nucleic Acids Res.* **39**, 8281–8290 (2011).

[9] Gapsys, V., Michielssens, S., Peters, J., de Groot, B. & Leonov, H. Calculation of binding free energies. In Kukol, A. (ed.) *Molecular Modeling of Proteins*, vol. 1215 of *Methods in Molecular Biology*, 173–209 (Springer New York, 2015).

[10] Auer, M. *et al.* High-yield expression and functional analysis of *Escherichia coli* glycerol-3-phosphate transporter. *Biochemistry* **40**, 6628–6635 (2001).

[11] Law, C. J. *et al.* Salt-bridge dynamics control substrate-induced conformational change in the membrane transporter GlpT. *J. Mol. Biol.* **378**, 828–839 (2008).

[12] Olsson, M. H., Søndergaard, C. R., Rostkowski, M. & Jensen, J. H. Propka3: consistent treatment of internal and surface residues in empirical pKa predictions. *J. Chem. Theor. Comp.* **7**, 525–537 (2011).

[13] Søndergaard, C. R., Olsson, M. H., Rostkowski, M. & Jensen, J. H. Improved treatment of ligands and coupling effects in empirical calculation and rationalization of pKa values. *J. Chem. Theor. Comp.* **7**, 2284–2295 (2011).

[14] Chen, V. B. *et al.* MolProbity: all-atom structure validation for macromolecular crystallography. *Acta Cryst. D* **66**, 12–21 (2010).

[15] Ensing, B., Laio, A., Parrinello, M. & Klein, M. L. A recipe for the computation of the free energy barrier and the lowest free energy path of concerted reactions. *J. Phys. Chem. B* **109**, 6676–6687 (2005).

[16] Law, C. J., Enkavi, G., Wang, D.-N. & Tajkhorshid, E. Structural basis of substrate selectivity in the glycerol-3-phosphate:phosphate antiporter GlpT. *Biophys. J.* **97**, 1346–1353 (2009).

[17] Enkavi, G. & Tajkhorshid, E. Simulation of spontaneous substrate binding revealing the binding pathway and mechanism and initial conformational response of GlpT. *Biochemistry* **49**, 1105–1114 (2010).

[18] Moradi, M. & Tajkhorshid, E. Computational recipe for efficient description of large-scale conformational changes in biomolecular systems. *J. Chem. Theor. Comp.* **10**, 2866–2880 (2014).

[19] Schlitter, J., Engels, M., Krüger, P., Jacoby, E. & Wollmer, A. Targeted molecular dynamics simulation of conformational change — application to the T $\leftrightarrow$ R transition in insulin. *Mol. Sim.* **10**, 291–308 (1993).

[20] Shenfeld, D. K., Xu, H., Eastwood, M. P., Dror, R. O. & Shaw, D. E. Minimizing thermodynamic length to select intermediate states for free-energy calculations and replica-exchange simulations. *Phys. Rev. E* **80**, 046705 (2009).

[21] Forrest, L. R. *et al.* Mechanism for alternating access in neurotransmitter transporters. *Proc. Natl. Acad. Sci. USA* **105**, 10338–10343 (2008).

[22] Crisman, T. J., Qu, S., Kanner, B. I. & Forrest, L. R. Inward-facing conformation of glutamate transporters as revealed by their inverted-topology structural repeats. *Proc. Natl. Acad. Sci. USA* **106**, 20752–20757 (2009).

[23] Radestock, S. & Forrest, L. R. The Alternating-Access mechanism of MFS transporters arises from Inverted-Topology repeats. *J. Mol. Biol.* **407**, 698–715 (2011).

[24] Lingenheil, M., Denschlag, R., Mathias, G. & Tavan, P. Efficiency of exchange schemes in replica exchange. *Chem. Phys. Lett.* **478**, 80–84 (2009).

[25] Hansford, D. Chapter 4 - beézier techniques. In Farin, G., Hoschek, J. & Kim, M.-S. (eds.) *Handbook of Computer Aided Geometric Design*, 75–109 (North-Holland, 2002).
